# Current and projected flood exposure for Alaska coastal communities

**DOI:** 10.1038/s41598-024-58270-w

**Published:** 2024-04-02

**Authors:** Richard M. Buzard, Christopher V. Maio, Li H. Erikson, Jacquelyn R. Overbeck, Nicole E. M. Kinsman, Benjamin M. Jones

**Affiliations:** 1 Department of Geosciences, University of Alaska Fairbanks, Fairbanks, AK 99775 USA; 2 U.S. Geological Survey, Pacific Coastal and Marine Science Center, Santa Cruz, CA 95060 USA; 3 Office for Coastal Management, National Oceanic and Atmospheric Administration, Anchorage, AK 99513 USA; 4 National Weather Service, National Oceanic and Atmospheric Administration, Anchorage, AK 99513 USA; 5 Institute of Northern Engineering, University of Alaska Fairbanks, Fairbanks, AK 99775 USA

**Keywords:** Natural hazards, Climate change

## Abstract

Globally, coastal communities experience flood hazards that are projected to worsen from climate change and sea level rise. The 100-year floodplain or record flood are commonly used to identify risk areas for planning purposes. Remote communities often lack measured flood elevations and require innovative approaches to estimate flood elevations. This study employs observation-based methods to estimate the record flood elevation in Alaska communities and compares results to elevation models, infrastructure locations, and sea level rise projections. In 46 analyzed communities, 22% of structures are located within the record floodplain. With sea level rise projections, this estimate increases to 30–37% of structures by 2100 if structures remain in the same location. Flood exposure is highest in western Alaska. Sea level rise projections suggest northern Alaska will see similar flood exposure levels by 2100 as currently experienced in western Alaska. This evaluation of record flood height, category, and history can be incorporated into hazard planning documents, providing more context for coastal flood exposure than previously existed for Alaska. This basic flood exposure method is transferable to other areas with similar mapping challenges. Identifying current and projected hazardous zones is essential to avoid unintentional development in floodplains and improve long-term safety.

## Introduction

Approximately 11% of people in coastal countries are located in low-elevation coastal zones^1^, and 23% of the global population is estimated to be in a 100 year floodplain^2^. Coastal zones provide an abundance of resources and are critical for shipping and trade. Coastlines are also subject to periodic flooding that endangers people and damages resources and infrastructure. Disaster risk management involves balancing society and hazards, a concept categorized into three groups: hazards, exposure, and vulnerability^3^.

Flooding is a natural process that can be hazardous. When people, resources, and assets are exposed to flooding, it becomes a disaster. Some exposed groups or assets are more vulnerable than others, such as having little capacity to mitigate a hazard or limited options to reside outside a hazard-prone area^4^. Exposure to flooding is increasing globally: from 2000 to 2015, Tellman et al.^5^ estimate the global population residing in floodplains grew by 20–24%. The global increase in disaster loss is more closely correlated with increased exposure and vulnerability than an increase in the frequency or magnitude of natural hazards^5,6^. Continued development in hazard-prone areas will only lead to worse disasters that are exacerbated by the negative aspects of accelerating warming and climate change^6^. However, disaster risk can be reduced by mitigation and adaptation strategies, such as identification and regulation of hazard zones^7^.

Climate change is acutely felt in northern regions where warming is faster than the global average and is destabilizing cryosphere processes that societies rely on^6^. In the Arctic, the three risk variables (hazard, exposure, and vulnerability) often overlap spatially, resulting in more frequent and severe disasters^8,9^. In addition, climate projections indicate that many critical changes have yet to be fully realized^6,9^: rising air and sea temperatures^6^, sea level rise^10^, sea ice decline^11^, storminess^12,13^, erosion^14,15^, permafrost thaw^14^, and barrier island dynamism^16^ are anticipated to accelerate in the coming decades. As an example of how these translate to disasters, ocean warming is speculated to have contributed to recent record flooding in Alaska^17^. In September 2022, Typhoon Merbok formed in record-high temperature waters in the west-Pacific where the water was historically too cold to regularly form typhoons^17^. Merbok remnants entered the Bering Sea and made landfall in Norton Sound, bringing record flooding to several communities^17^. The storm downed power lines, spilled fuel tanks, caused considerable erosion, floated buildings off their foundations, and damaged critical subsistence equipment^18^. While Merbok demonstrates the potential for climate change to increase flood hazards, it is also important to recognize the long-standing social aspects leaving communities exposed to flooding. Most of the communities flooded by Merbok have experienced several other storm-driven major floods in the last 50–100 years^19–21^, yet they still do not have a mapped floodplain or estimated record flood elevation to guide safer development^22^. Given the ongoing and potentially increasing flood disasters, communities need comprehensive flood risk assessments and hazard zone mapping that incorporate climate models^23,24^.

This study investigates coastal flood hazards and exposure in 63 Alaska communities located on the Bering, Chukchi, and Beaufort Seas. The approach focused on (1) developing a coastal flood database using written accounts and observations; (2) identifying record flood events and estimating the elevation using a combination of observations and remote sensing techniques; (3) estimating current flood exposure by comparing record flooding to topography and infrastructure locations; and (4) projecting future flood exposure using global projections of relative sea level rise.

## Results

Results are presented for each of the four objectives. Community-specific results are given for objectives 1 and 2 (Flood History Database; Record Floods and Categories). Objectives 3 and 4 (Current and Projected Flood Exposure) are presented using regional summaries. There are 63 coastal communities in the study area, although certain analyses were not possible in some communities due to data limitations. See the Methods section and Supplementals for more details about available datasets, analysis, and results visualization decisions.

### Flood history database

**Figure 1:**
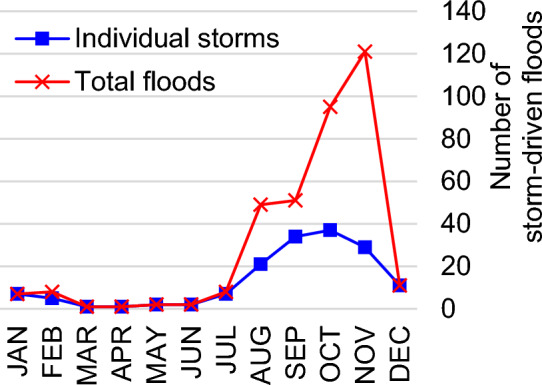
Monthly storm-driven flood events. Individual storms (blue squares, defined as a single storm even if it affected multiple communities) occur more often from August through November, peaking in October. The total number of communities flooded (red X) is greatest in November. Storm-driven flooding is rare from March through June.

The Flood History Database provides an inventory of all recorded coastal flood events in western and northern Alaska based on the available sources analyzed. Database entries are community-specific, so a single flood affecting multiple communities will generate multiple entries. Attributes include the flood date, flood/storm event identification number, communities impacted, severity, estimated height, photos, and written descriptions. Records go back to 1887, but there are far more observations recorded in recent decades. Contributions are ongoing, and by June 2023 the database had 448 entries for 55 of the 63 coastal communities investigated in this study. The remaining 8 communities appear to not experience flood hazards (Supplemental 2). There were 382 entries for storm-driven floods, 48 for ice jams, 3 for rainfall, snowmelt, and/or spring runoff, and 15 with unknown/unspecified cause. Of the 382 storm-driven floods, 176 (46%) were individual storms large enough to affect multiple communities (in other words, 54% of storm-driven floods reportedly only impacted 1 community). Overall, 76% of storm-driven floods occurred in fall (September through November; Fig. 1). Most storms occurred in October, yet most communities were flooded in November. In other words, storms are more frequent in October but more widespread and severe in November. The number of floods declined steeply into December and remained low until August.

### Record floods and categories

The highest storm-driven flood elevations for each community are listed in Table 1. These record flood elevations estimate the total water level of widespread flooding, approximately representing the sum of tide, surge, and wave setup. For some communities on spits, a second value is provided to estimate only the combined tide and surge (“storm tide”), which represents the maximum water level for the sheltered parts of their coastline. Elevations are given in meters above local mean higher high water (MHHW) and the North American Vertical Datum of 1988 (NAVD 88 [GEOID12B]). Uncertainty is estimated at a 95% confidence interval. Of 63 communities analyzed, 8 had inconsequential flooding and an additional 9 had minor to inconsequential flooding with insufficient descriptions or datasets to estimate the record flood elevation. Record flood estimates are made for the remaining 46 communities.

**Table 1:** Estimated total water level and/or storm tide elevations of the record storm-driven floods.

Community	Date	Flood height (m local MHHW)	Flood height (m NAVD 88)	95% Confidence interval (m)	NWS Flood impact category
Alakanuk	1974-NOV-10-11	1.98	4.49	0.15	Major
Brevig Mission	1974-NOV-10-11	3.05	4.19	0.50	Moderate
Chefornak	2012-OCT-04	1.10	3.17	0.40	Minor
Chevak	2022-SEP-17	1.86	3.91	0.32	Major
Chignik Bay	1948-OCT	1.55	3.79	0.15	Major
Clark’s Point	1929-NOV	2.19	6.20	0.34	Major
Deering	1973-NOV-10-15	3.08	4.55	0.30	Major
Diomede	1990-NOV-18	3.11	4.23	0.61	Major
Ekuk	1969	2.19	6.20	0.34	Major
Elim	1945-OCT-28	4.79	6.39	0.30	Moderate
Golovin	1945-OCT-28	3.66	5.25	0.61	Major
Goodnews Bay	2011-NOV-10-11	3.41	5.50	0.15	Major
Hooper Bay	2005-SEP-22	2.35	4.41	0.43	Major
Kaktovik	1964	1.66	2.86	0.30	Minor
Kipnuk	2016-OCT-28	1.37	3.73	0.06	Major
Kivalina	1970-SEP	2.68 *1.83	3.75 *2.90	0.37 *0.55	Minor
Kongiganak	Unknown	3.14	6.03	0.61	Minor
Kotlik	1974-NOV-10-11	2.35	4.25	0.12	Major
Kotzebue	2012-AUG-25	1.83 *1.10	3.05 *2.33	0.48 *0.33	Major
Koyuk	1913-OCT-05	3.99	5.74	0.61	Major
Kwigillingok	2012-OCT-04	1.31	4.20	0.34	Major
Mekoryuk	1974-NOV-10-11	1.80	3.9	0.6	None
Napakiak	1990-AUG-17	2.10	5.22	0.34	Major
Newtok	2005-SEP-22-23	1.8	3.9	0.6	Moderate
Nightmute	2011-NOV-10-11	0.58	2.90	0.18	Minor
Nome	1913-OCT-05	3.81	5.06	0.67	Major
Nunam Iqua	2013-NOV-05	2.59	4.57	0.30	Major
Platinum	1979-NOV-09	2.62	5.27	0.30	Moderate
Point Hope	1893-OCT-13	3.60	4.74	0.25	Major
Quinhagak	1989-AUG-17	2.68	5.66	0.40	Moderate
Saint Michael	1974-NOV-10-11	3.96	5.41	0.24	Minor
Saint Paul	1966-DEC-25	3.7	3.8	0.6	Moderate
Scammon Bay	1976-AUG	2.4	4.5	0.6	Major
Shaktoolik	1960-OCT-02	5.06 *2.71	6.91 *4.57	0.18 *0.29	Major
Shishmaref	1973-NOV-09-10	2.29 *1.68	3.35 *2.74	0.34 *0.34	Moderate
Stebbins	1960-OCT-02-03	4.33	5.79	0.30	Major
Teller	1913-OCT-05	2.9	4.0	0.6	Major
Togiak	1964-OCT	2.3	5.0	0.4	Major
Toksook Bay	1995-OCT-28	1.86	4.18	0.34	Minor
Tuntutuliak	1990-AUG-17	0.6	3.4	0.3	Major
Tununak	2011-NOV-08-11	2.0	4.3	0.4	Minor
Umkumiut	1995-OCT-28	1.86	4.18	0.61	Minor
Unalakleet	1965-NOV-05	4.5 *2.9	6.1 *4.5	0.6 *0.6	Major
Utqiaġvik	1963-OCT-03	3.54	4.57	0.30	Major
Wainwright	1963-OCT-03	3.51	4.54	0.69	Minor
Wales	1974-NOV-10-11	4.11 *3.51	5.22 *4.00	0.61 *0.61	Moderate

Values are listed as meters above local tidal and orthometric datums. Uncertainty is estimated at a 95% confidence interval.

*denote estimates that are only tide and surge for sheltered coastlines. Flood categories refer to Alaska National Weather Service guidelines^22^. Community-specific methodology and contributing sources are found in Supplemental 2.

**Figure 2:**
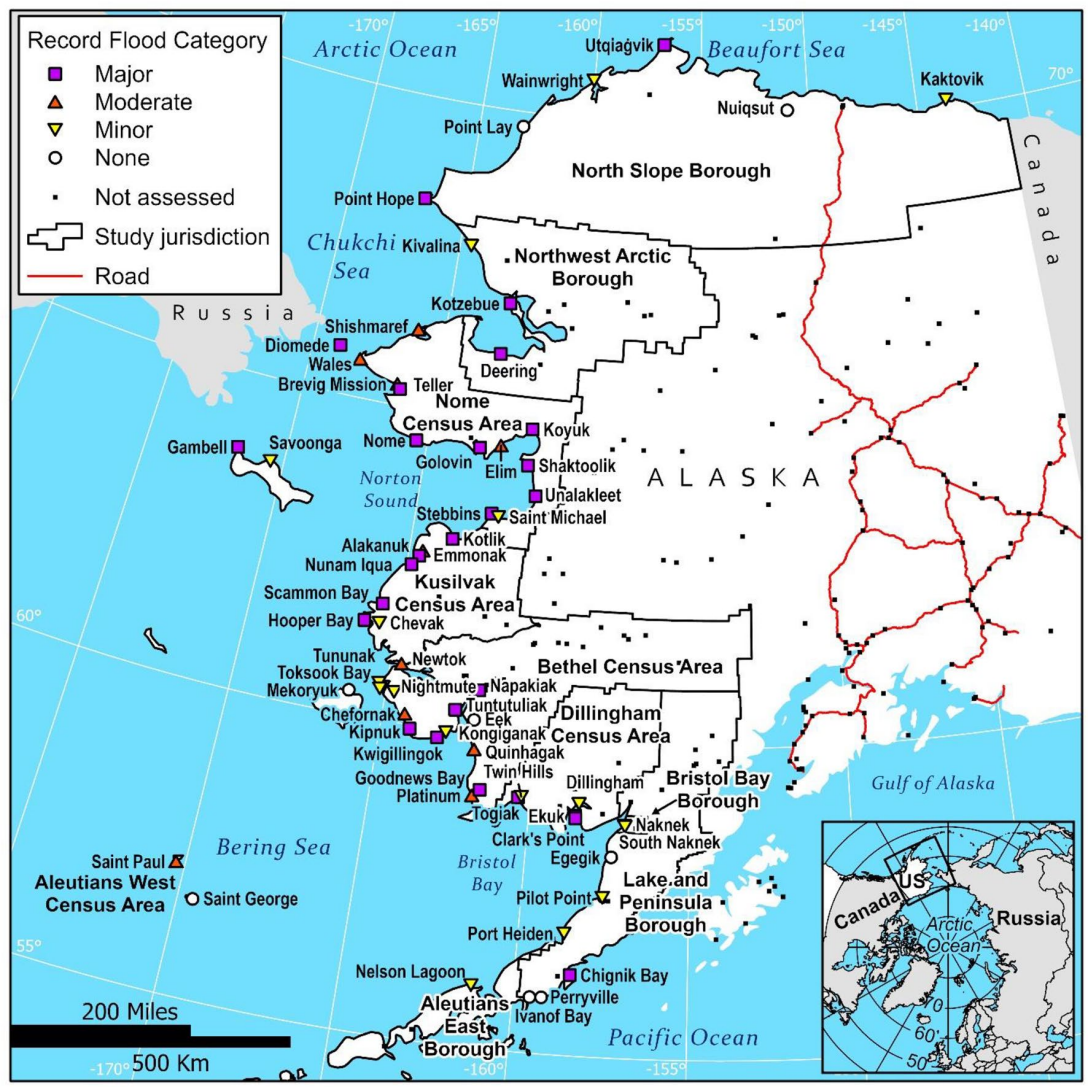
Map of highest storm-driven flood impact category in 63 Alaska coastal communities for flood events from 1887 to 2022. Categories are major (purple square), moderate (orange triangle pointed up), minor (yellow triangle pointed down), and no known flooding (white circle). Communities not assessed are black dots. The road system (red) does not reach any of these remote locations. Forty-six of these communities (listed in Table 1) also have elevation models and record flood descriptions that allowed us to conduct a flood exposure analysis.

The Alaska National Weather Service (NWS) flood impact category is estimated for the record flood in all 63 communities (Tables 1 and 2; Fig. 2). Flood categories represent the severity of flooding for the community when it occurred. Since flood categories are determined through descriptive accounts, they can be estimated for all communities even when record flood elevations are not estimated. Of the 63 communities analyzed, 59% have experienced moderate to major flooding and only 13% experience no consequential flooding (Tables 1 and 2; Fig. 2). The Nome Census Area has the most communities that experience storm-driven flooding, especially major flooding. Over half of the coastal communities in the areas of Nome, Kusilvak, and Dillingham jurisdictions have experienced major flooding. The Bethel Census Area also has several communities that experience major flooding.

**Table 2:** Number of communities in each jurisdiction that have experienced a given flood category in a record flood.

Jurisdiction	n	None	Minor	Moderate	Major
North Slope Borough	6	2	2	0	2
Northwest Arctic Borough	3	0	1	0	2
Nome Census Area	15	0	2	4	9
Kusilvak Census Area	7	0	1	1	5
Bethel Census Area	16	2	5	4	5
Dillingham Census Area	5	0	2	0	3
Lake and Peninsula Borough*	6	3	2	0	1
Bristol Bay Borough	2	0	2	0	0
Aleutians East Borough*	1	0	1	0	0
Aleutians West Census Area*	2	1	0	1	0
Total	63	8	18	10	27
Percent of total	100%	13%	29%	16%	43%

Jurisdictions with asterisks (*) do not include every coastal community.

### Current and projected flood exposure

Flood exposure is estimated by comparing record flood elevations to the ground elevation of existing structures. The record flood elevation is used to define the “current floodplain” (as of approximately the year 2020 based on this report’s flood history database). Projected flood exposure is estimated by increasing the record flood elevation by relative sea level rise (RSLR) projections using Alaska’s results from global mean sea level (GMSL) scenarios of 0.5 m and 1.0 m by 2100^10^. First floor elevations of structures were unknown, and most structures are elevated above ground level, so the following metrics represent water depth at structure locations but not necessarily inside structures. Flood exposure results are examined study-wide and per jurisdiction.

**Figure 3:**
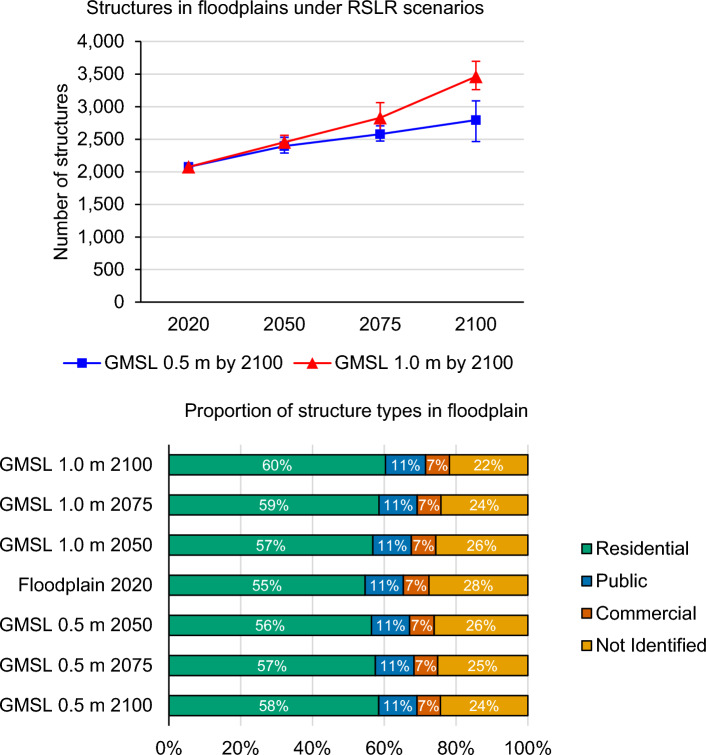
(Top) Number of structures located within the record floodplain (as of approximately 2020) and in the future based on RSLR projections under GMSL scenario 0.5 m by 2100 (blue squares) and 1.0 m by 2100 (red triangles). (Bottom) Proportion of structure types found within current floodplains and with RSLR. Residential structures (green) are most common, followed by unidentified (yellow), public (blue), and commercial (red). Proportions do not change much across different RSLR scenarios.

Of the 46 communities analyzed for flood exposure in western and northern Alaska, 2076 of 9273 coastal structures (22%) are located within a floodplain (Fig. 3). At least 55% of these are residences, although another 28% are residence-sized or larger structures that are not identified and may be mostly residences. Under GMSL scenario 0.5 m by 2100, 2794 structures (30%) would be within a floodplain by 2100. Under GMSL scenario 1.0 m by 2100, the previous value is exceeded by the year 2075 and 3461 structures (37%) would be within a floodplain by 2100. In all scenarios, flood expansion leads to a slightly higher proportion of residences within the floodplain than other building types.

**Figure 4:**
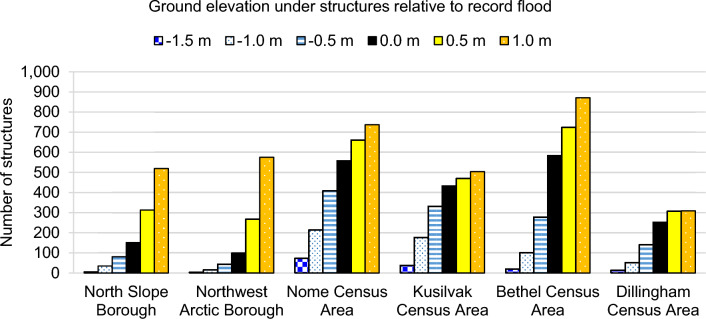
Number of structures with ground elevation above or below the record flood elevation in each jurisdiction. The structures within the floodplain (0.0 m) are shown by the solid black bar. Blue-scale bars show the structures on ground below the record flood elevation, resulting in negative values that represent depth (e.g., − 1.0 m means water is 1.0 m deep at those structures). Yellow-scale bars show the structures on ground just above the record flood elevation, which can be compared to RSLR rates to estimate projected flood exposure. Jurisdictions are listed from north to south.

**Figure 5:**
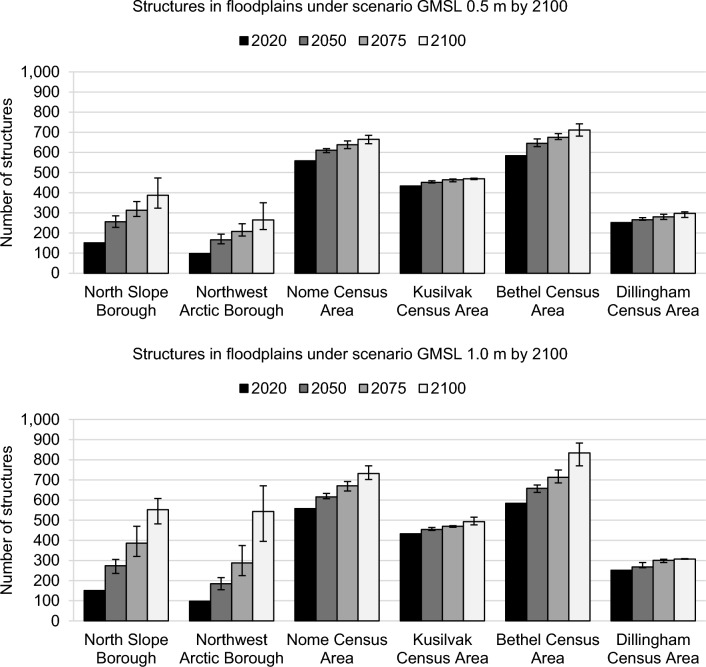
Total number of structures within the record floodplain over time for each jurisdiction under GMSL scenarios of 0.5 m and 1.0 m by 2100 (top and bottom graphs, respectively). The Bethel Census Area has the greatest number of structures in a floodplain for all time steps in both projections, closely followed by the Nome Census Area.

There are six jurisdictions in the study area where flood extent analysis was possible (Fig. 4). Of these, the Nome, Kusilvak, and Bethel jurisdictions have 76% of exposed structures in the study, and the greatest number of structures that would experience depths up to 1.5 m during a record flood (approximately the depth needed to flood the average residence). The North Slope and Northwest Arctic Boroughs have the lowest number of structures within a floodplain, but considerably more structures on ground only 1.0 m above the floodplain (Fig. 4). These jurisdictions also have the fastest projected RSLR (Supplemental 5). This combination results in the largest increase in projected flood exposure (Fig. 5). Under GMSL 0.5 m, the Nome, Kusilvak, and Bethel jurisdictions maintain the most structures in floodplains. Under GMSL 1.0 m, The Nome and Bethel areas continue to have the greatest number of structures in floodplains, but by 2100 the North Slope and Northwest Arctic Boroughs are projected to reach similar flood exposure as the southern jurisdictions currently experience. Combined, the North Slope and Northwest Arctic Boroughs are projected to have 162–340% more structures in floodplains by 2100. The Dillingham Census Area communities are split between lowland and highland areas, so the number of structures within the floodplain does not change much due to RSLR once the lowland area is entirely a floodplain. RSLR projections are listed per jurisdiction in Supplemental 5.

Of the 46 communities analyzed, 38 (83%) have at least one percent of structures within the floodplain and 15 communities (33%) have more than half of all structures within the floodplain (Table 3; the current floodplain is represented by > 0.0 m in 2020). There are 4 communities where more than half of all structures would experience at least 0.9 m depth in a record flood, enough to total vehicles other than light trucks^25^. There are 15 communities where some structures would experience 1.5 m depth, nearly reaching the mean first floor height of commonly flooded communities (Supplemental 4). Table 3 lists changes in flood exposure due to RSLR. Under the GMSL scenario 0.5 m by 2100, 42 communities (91%) would have at least one percent of structures within the floodplain by 2100 and 19 communities (41%) would have more than half of all structures within the floodplain by 2100. Under the GMSL scenario 1.0 m by 2100, the previously stated values would be achieved by 2075, with flooding reaching 0.3 m deeper by 2100.

**Table 3:**
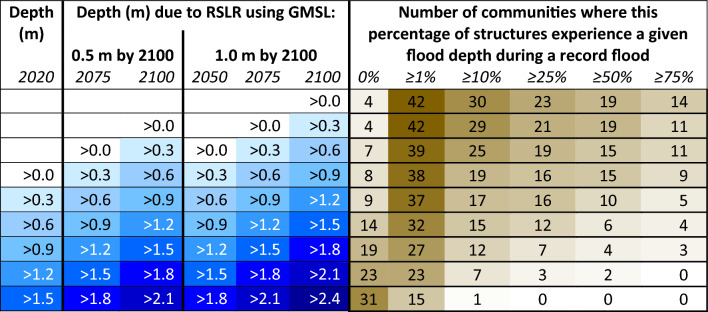
Comparison of flood depth and extent due to record flooding and RSLR. The first column shows depths in the current floodplain.

The brown columns show the percentage of structures experiencing given depths during a record flood, and each number is the count of communities meeting the row and column criteria. For example, the cells for the 2020 record depth of > 0.3 m and ≥ 10% flooded result in 17 communities, meaning there are 17 communities where at least 10% of structures experience greater than 0.3 m depth during a record flood. The depths due to RSLR show how these values are projected to change over time. For example, under the *GMSL 0.5 m by 2100* scenario, by 2100 there are 25 communities where ≥ 10% of structures are flooded to a depth of > 0.3 m during a record flood. The 0% category means no structures experience the given flood depth, so there are 4 communities analyzed that have no structures projected to be in a floodplain by 2100.

## Discussion

The results of this study demonstrate the existence of substantial coastal flood exposure present in Arctic Alaska that is projected to worsen considerably with sea level rise. The following discussion outlines possible root causes for current and projected exposure. The limitations of this basic-level flood exposure are discussed alongside suggestions to improve flood risk studies in Alaska and abroad.

### Flood prevalence and causes of exposure

The study region has a disproportionately high percentage of the population living in floodplains. To put flood exposure results into greater context, comparisons are made to country and global levels. This is a large, sparsely populated area. The 6 jurisdictions of the flood exposure analysis have an area of 514 km^2^ (30% of Alaska), which is larger than all U.S. states except Texas, and larger than 75% of all countries. However, there is a relatively low population of 60,800 people, and only 31,200 people in the 46 analyzed communities^26^. Twelve of the twenty-two percent of structures in the floodplain are residences, and another 6% are residence-sized, unidentified structures. From these figures, we estimate 12–18% of the population of the 46 studied communities (3700–5600 people) are residing in a coastal floodplain. This is 10–15% of the population of the 63 coastal communities studied. In the United States, approximately 3–5% of the population is estimated to live in coastal floodplains^27–29^. Communities in western and northern Alaska have more than double the national proportion of people living in coastal floodplains. However, the total number of people in floodplains is far less than any other state^30^.

Flood exposure is the overlap of natural hazards with the built environment, and flood exposure is more often driven by development in floodplains than expansion of floodplains due to climate change^5,6^. This observation also appears to be at the root of why many coastal communities in Alaska experience major flood disasters. The U.S. Government Accountability Office^31^ explains, “In many cases, Native villages occupy their current precarious locations because the federal government built schools in what were seasonal encampments and mandated that Alaska Native children attend those schools.” These seasonal encampments were often found in low-lying areas and on spits within the floodplain^32,33^. Additionally, for over a century after the purchase of Alaska in 1867, Indigenous peoples in Alaska had little to no land ownership rights and were not controlling decisions over the permanent location of their community^32^. The school system and other aspects of colonization (i.e. religious institutions, canneries, epidemics, and fuel access) led to the consolidation of communities into larger settlements^32,34,35^. With the passing of the Alaska Native Claims Settlement Act of 1971, communities gained more autonomy over development^32^. However, the number and cost of structures in floodplains grew substantially during the interim century. Some communities partially or fully relocated from flood and erosion hazards in the early to mid-1900s^36^. Now, communities are too large to be relocated easily and have relied on government assistance to mitigate or adapt to ongoing hazards^19,31,37^. Alaska Native villages are often framed as being more acutely at risk due to climate change due to remoteness, reliance on subsistence resources, and the destabilizing landscape due to permafrost thaw and sea ice decline^19,38^. However, exposure to natural hazards in Indigenous communities is largely driven by the long-standing influence of colonization and the suppression of autonomy, especially regarding land ownership and decision-making^38,39^. These factors have led to the relatively high proportion of Alaska’s rural communities, majorly Alaska Native villages, residing in floodplains.

The low population of rural Alaska communities is an obstacle to addressing flood exposure with government assistance. The U.S. Government Accountability Office^31^ explains how these small, rural communities face systemic obstacles to obtain federal assistance due to population size thresholds needed for competitive grant programs. There are also barriers to mitigation funding because of tribal and subsistence attributes, such as cost-share requirements, cost–benefit analyses, disaster declaration requirements, and some programs finding federally recognized tribes ineligible to apply^31^. Fortunately, these long-standing barriers are beginning to be recognized and addressed by federal agencies.

Historical development in floodplains and systemic barriers to mitigation contributed significantly to the current flood exposure in Alaska and will continue to influence future exposure, in addition to climate change. This study estimates substantial increases in flood exposure due to RSLR under the assumption that communities maintain development in the same location. Other major contributors not examined are permafrost thaw-driven subsidence and increased storm activity due to sea ice decline, warmer air and warmer water^8,13,40,41^. Given the current prevalence of hazards and projected warming in the Arctic, now is a critical time to reevaluate these paradigms that have led to the current state of disaster loss^6,31^.

### Study limitations and recommendations

This study performs a basic flood exposure analysis^25^ using record flood levels, elevation models, structure locations, and sea level rise projections. An advanced, local-level analysis depends on more datasets that are available for several communities. The advanced analysis would include an empirical model of the flood hazard, overland flow, relevant projected environmental changes, and a more detailed and locally relevant infrastructure inventory. Traditionally, the 100-year flood elevation is computed using the frequency and magnitude of flooding. Statistical water level parameters can be computed from long-term water level sensors and global climate models^42–44^. Converting offshore models to coastal models and overland flow requires wave modeling and a connection between the tidal datum, elevation models, and bathymetry^43^. Overland flow models are further supported by surface geology and vegetation cover maps. In high-latitude coastal zones, studies often limit modeling periods depending on sea ice coverage, as ice can both limit the fetch available for winds to generate surge and also increase water levels^13,45^. Landscapes and the climate are dynamic and changing, so future-looking flood models must incorporate sea level rise^10^, changing storm climate^44^, sea ice decline, permafrost thaw subsidence, and erosion^23^. A major limitation of this study's sea level rise projections are that only two sea level rise measurement nodes were available for western and northern Alaska^10^, making it challenging to confidently project or assess sea level rise estimates. Vertical land movement measurements are more widely available^46^. However, in areas of ice-rich permafrost, warming (more thawing degree days) could increase subsidence rates beyond historical levels^47,48^. Many of these datasets and models are becoming more commonly available for Alaska’s rural communities, allowing for advanced flood hazard and overland flow models. A keystone to support such efforts is the inclusion of validation data, namely flood observations made by residents of communities^22^.

The basic flood analysis method defined by The U.S. Federal Emergency Management Agency^25^ suggests using building area, building value, transportation systems, utilities, vehicles, and basic economic data. An advanced analysis incorporates structure type, construction, first floor elevation, age, and contents. These parameters help identify how the flood depth and overland flow would affect individual structures and contents. The infrastructure inventory can be expanded to roadways and utilities if there are documented cases of the level of flooding required to damage them. For example, many communities have utility corridor systems (aboveground insulated corridors for water, sewer, and electric) that are periodically damaged during floods. There are several other infrastructure aspects unique to northern regions and rural, subsistence communities that will require special emphasis, such as damage to boardwalks, fuel tanks, and sewage lagoons. In addition, there are many small structures that house subsistence equipment that is expensive and difficult to replace^49^. These important structures and their contents could not be included in this report because they were typically not delineated in available structure data sources (Supplemental 4). Structures and property may also experience different levels of impact depending on inundation duration, flow velocity, and wave action. Detailed infrastructure data layers and flood interactions are necessary to better understand and model flood exposure.

### Vulnerability and special considerations

Vulnerability is the most challenging aspect of risk analysis to quantify, as it requires deeper knowledge of how hazards affect communities and the local and state capacity to support recovery. Cardona^50^ explains that vulnerability is the manifestation of the social construction of risk. For example, in small, remote, rural communities, a common vulnerability is the lack of redundancy in critical infrastructure. There is often only one power plant, water treatment system, and evacuation road. In northern regions, losing access to critical infrastructure during the winter can have life-threatening consequences. For individuals and households in Alaska, another unique and important factor of vulnerability relates to the dependence on subsistence-related equipment, vehicles, and food. These tools are necessary for subsistence activities, an essential practice in this region. Vehicles are not commonly insured and cost much more to replace than retail prices due to shipping. Even if equipment can be replaced, this can take a long time and the individuals or communities will not be able to do vital subsistence activities in the interim. Advanced warning allows people to move subsistence equipment to high ground and reduce flood impacts^51^. However, advanced warning can only be given to areas where forecasters are aware of the risks, an identification that requires accurate accounting of risk factors.

The following example illustrates the importance of incorporating remote and subsistence variables into risk analyses to accurately reflect the situations in rural Arctic communities. While this study was being conducted, the storm remnant of typhoon Merbok brought record-breaking major flooding to the Yukon-Kuskokwim Delta and Norton Sound. Many communities had widespread flooding under and inside structures. Chevak, a community built on a bluff above the floodplain, was presumed safe from flooding. However, boats and subsistence equipment were being stored in a nearby lowland that became submerged by up to 2 m of flowing water during the peak of the storm. Since this exposed area was not known by forecasters, residents were not advised to move the equipment to high ground. The flood destroyed nearly 90 of the 100 boats in Chevak that are used for fishing and hunting^49^. Boats can cost more than $20,000 each to purchase and ship to this region^52^. This is over half of the annual median household income for Chevak ($37,975), in the jurisdiction with the lowest income of the state^53^. In addition, the flood destroyed and carried away sheds filled with equipment like life jackets, gasoline, tackle, and subsistence gear. The winds also knocked out power to half of the community for three days, thawing freezers of food stores for the winter. USACE^54^ estimated subsistence meat is valued at 5 times the cost of imported meat due to shipping costs. Schwing^49^ summarizes, “People have lost not only the food they’ve already gathered, but also their means to replace it.” The resulting disaster in Chevak was due to several factors relating to the major flood, the unrecognized exposure leading to inadequate warning or preparation, and vulnerabilities common to remote Arctic communities. While the disaster event is over, there are long-lasting negative impacts due to the loss of equipment and resources, as well as delayed aid, that perpetuate an increased state of vulnerability^55^. These impacts can be lessened or avoided with timely and accurate forecasts, floodplain maps, exposure analyses that incorporate community-relevant assets, and new development outside of floodplains.

### Conclusions

Coastal flood disasters pose a considerable challenge globally, and especially so in rural Alaska. This study compiled a database of floods for over 60 coastal communities of northern and western Alaska. We identified community-specific record floods, finding that over half of the communities have experienced moderate to major flooding. Record flood elevations were estimated for 46 of the 63 communities, then compared to elevation models and infrastructure to compute flood exposure. At least 22% of their structures are currently in floodplains. Sea level rise projection scenarios suggest this will increase to 30–37% of structures by 2100 if communities continue developing in the same at-risk location. Jurisdictions from the Yukon-Kuskokwim Delta to Norton Sound currently have the most structures in floodplains. Jurisdictions farther north are projected to experience the greatest increase in flood exposure, with flood waters reaching developed areas that have historically been above the floodplain. Flood record and sea level rise results can be used with available topographic datasets to map the current and projected floodplain in community adaptation plans. Recommendations are provided to improve community-specific flood exposure analyses by incorporating important metrics unique to rural, Arctic communities reliant on subsistence resources.

## Methods

We compiled flood information, estimated record flood elevation, and evaluated regional coastal flood exposure of Arctic Alaska coastal communities. The number of communities included in each analysis phase vary depending on available data for each objective (Supplementals 1, 2, 3). Any community with a record of flooding is included in the flood database. However, there must be enough detailed information about events to estimate the record flood elevation. Flood exposure further requires a digital elevation model (DEM; Supplemental 1), a connection between the tidal and orthometric datums, and infrastructure spatial data.

### Compiling the flood database

A database was created to list all known coastal flood events in western and northern Alaska. Most communities do not have long-term water level measurements, so the flood event entries are most often written descriptions. Sources common to many communities include NOAA Climatological Data reports (1915–1980) and Storm Data reports (1959-present) available from https://www.ncdc.noaa.gov; investigations by USACE (circa 1960s to present) available from https://www.poa.usace.army.mil/About/Offices/Engineering/Floodplain-Management/; hazard mitigation plans and community plans available from the State of Alaska (https://www.commerce.alaska.gov/); other government reports; and scientific investigations.

The flood database contains, at a minimum, the impacted community, date, and the source description (e.g., Koyuk, October 2–3, 1963, “Highest in local memory. A cabin near the intersection of First Avenue and Tamarack Street had 1 foot of water on the first floor during this storm,” from Koyuk’s 2014 hazard mitigation plan). Some floods have been analyzed to determine the NWS flood category. Some floods also have an estimated high water elevation in NAVD 88 (GEOID12B as of this study date) and relative to the local tidal datum if available. Conversions between NAVD 88 and tidal datums are made using the Alaska Tidal Datum Portal https://dggs.alaska.gov/hazards/coastal/ak-tidal-datum-portal.html. The flood database is hosted online at https://water-level-watch.portal.aoos.org/ (titled “Flood Events” in the catalog of data layers). Contributions are ongoing by project partners.

When possible, floods were categorized as minor, moderate, or major, following National Weather Service guidance^22^. These categories are based on described impacts at the time the flood occurred, not the impacts that would occur if the flood happened with infrastructure present today. Flood categories focus on fixed infrastructure (such as buildings and roads) and societal impacts (such as loss of electricity or triggering evacuations). Erosion is a significant result of flooding, but erosion is not counted as a flood impact for this analysis. Isolating flood and erosion impacts is necessary to improve combined threat assessments so that erosion impacts are not scored twice^19^. Separate studies exist to quantify erosion impacts^56^.

### Estimating the record flood elevation

Flood hazards are traditionally computed using water level data to compute return periods, with the 100-year flood used as the standard to map the floodplain. Water level data is notoriously sparse in the study area. Following Federal Emergency Management Agency^25,57^ and U.S. Army Corps of Engineers^19^ (USACE) guidance, we used the record flood elevation as a proxy for the 100-year flood. The record flood for each community is identified from the flood database. Many communities have a record flood estimate made by USACE and the Alaska Division of Community and Regional Affairs. However, the estimates were typically made in the 1990s, and several places have experienced higher floods since. Recent experiences are often described with greater detail in hazard mitigation plans, but the elevation is usually not estimated in a comparable datum. We compare findings of previous investigations to more recently available sources to determine which flood event is the record. The record flood elevation is estimated using geodetic conversions and measuring high water mark elevations with DEMs^58^. If an estimate already exists, we examine the source data and rationale, as well as compare the result to observations and elevation models. This process helps resolve conflicting datum issues that often arise from the long-standing disconnect of tidal and geodetic datums.

Record flood estimates comprise tide, surge, and wave setup, but not wave runup. Some locations are alongshore spits with back-barrier lagoons or rivers. The ocean side of the spit experiences all water level components, but the backside experiences only tide and surge (storm tide)^59^. Where applicable, the storm tide water level is estimated separately. The data sources and methodology for each estimate are detailed in Supplementals 2 and 3.

### Representing exposure using structure datasets

The Intergovernmental Panel on Climate Change^6^ defines exposure as, "The presence of people; livelihoods; environmental services and resources; infrastructure; or economic, social, or cultural assets in places that could be adversely affected.” Structure datasets represent many of these exposure aspects (e.g. residences represent the presence of people, critical infrastructure represents livelihoods and services). For this study, structure location and size are used to estimate exposure. This data-limited approach constitutes a basic exposure analysis, so results are displayed at census-tract scale^25^.

Structure footprint datasets were collected from Buzard et al.^56^ and the North Slope Borough. In most communities, structures were originally digitized for Community Profile Maps in the mid-2000s (maps are available from the Alaska Division of Community and Regional Affairs website at https://www.commerce.alaska.gov/web/dcra/). Buzard et al.^56^ used recent imagery to update footprints. For most communities, structure datasets include classification of residential, commercial, and public structures. While smaller structures like garages, sheds, shipping containers, and fish smokehouses are particularly important for communities within the study area, they could not be included for regional comparison because they were not consistently digitized in the source datasets. We compared structure area statistics to determine that a 56 m^2^ threshold should be applied to remove small structures without significantly reducing the number of target structures (Supplemental 4).

### Current flood exposure

In the 2019 Alaska Statewide Threat Assessment^19^, flooding is defined as “… the inundation of infrastructure or the impassibility of airstrips and roads due to elevated water levels along a coast or river.” This study uses the same metric; flood exposure is determined by computing water depth at structures during a record flood. The record flood elevation is assumed to be static for each community. Some communities are on islands or spits with a sheltered side, and observations indicate water levels are lower than the exposed side during record flood events. In these locations, we use two static flood elevation estimates (exposed = tide + surge + wave setup; sheltered = tide + surge). The designation of sheltered or exposed coastline infrastructure is made depending on the flood extent history and existing topography that would impede exposed-coast flooding from reaching the sheltered side, such as a shore-parallel berm that exceeds the record flood elevation.

Digital elevation models (DEMs) were used to compute the ground elevation underneath structures. DEMs are available in each study location, but type and quality vary. Digital terrain models (DTMs) represent bare-earth and are ideal for floodplain mapping. Lidar-derived DTMs were available for 22 of the 48 communities analyzed. Almost all communities have Community Profile Maps with 0.6 m contour lines relative to a geodetic datum available from the Alaska Division of Community and Regional Affairs. In 3 communities where no DEM existed, we converted these contours to DTM rasters and assessed accuracy using global navigation satellite system survey data collected during earlier site visits. Most communities have digital surface models (DSMs) that represent the elevation of the highest feature, such as building roofs and the top of tall grass and shrubs. DSMs are less ideal for floodplain mapping than DTMs because water can flow underneath structures and through vegetation. We prioritize using DTMs over DSMs and consider DEM age and significant modifications to overland flow, such as raised elevations to serve as levees. DEM sources and statistics are listed in Supplemental 1.

Depth is calculated by comparing the record flood elevation to the ground elevation under structures. For DTMs, ground elevation is computed using the median elevation within the structure polygon. The median is preferred over the mean to reduce error from model noise (Supplemental 1). For DSMs, ground elevation is computed using the minimum elevation within 1 m of the polygon. This statistic is necessary to avoid the elevation of the structure, surrounding objects (stairs, grass/shrubs, miscellaneous property), and the sloping artifact common to DSMs at high-angle features. We assess accuracy by comparing a lidar-derived DTM to contour-derived DTMs, crewed aircraft-derived DSMs, and uncrewed aircraft-derived DSM in Supplemental 1. All sources were suitable to substitute the lidar DTM if needed.

### Projected flood exposure

Potential future flood exposure is projected using relative sea level rise (RSLR) projections. Sweet et al.^10^ project RSLR at several nodes across Alaska. Change is referenced to MSL in 1992, the mid-point of the 1983–2001 National Tidal Datum Epoch. Each community has a local tidal datum with MSL connected to orthometric elevation. This datum connection allows the RSLR results to be added to the record flood elevation and compared to DEMs. For each community, the average RSLR of their jurisdiction is added to the record flood elevation, which is then recomputed to estimate flood exposure of structures given RSLR. This process is run for projections to 2050, 2075, and 2100 using the median RSLR and 95% confidence intervals.

For this study, we selected two RSLR scenarios: GMSL of 0.5 m by 2100 and 1.0 m by 2100. These represent mid-range to slightly more extreme outcomes for the region across the range of possible global emissions scenarios^10^. RSLR scenarios depend on possible emission scenarios and resulting temperature change. The likelihood of these scenarios depends on the amount of emissions and associated warming. Total warming is projected to be between 1.5 and 5.0 °C higher than 1850–1900 average temperatures under low to very high emission scenarios, respectively. If temperatures are only raised by 2 °C in 2100, Sweet et al.^10^ estimate there is a 50% likelihood that global mean sea level (GMSL) will reach 0.5 m by 2100 and less than a 5% likelihood to reach 1.0 m. If temperatures are raised by 5 °C, there is a greater than 99% likelihood of reaching 0.5 m and 23% likelihood to reach 1.0 m.

### Supplementary Information

Supplementary Information.

## Data Availability

The flood database is hosted online at https://water-level-watch.portal.aoos.org/ (titled “Flood Events” in the catalog of data layers). Elevation model data source locations are described in Supplemental 1. Most elevation models are publicly available or available upon request from the source organization. Structure footprint shapefiles are available for download from https://doi.org/10.14509/30573. North Slope Borough community structure shapefiles were available upon request from the North Slope Borough Community Planning and Development Division. Relative sea level rise projections are available from Sweet et al.^10^. Conversions between tidal and orthometric datums can be computed using the Alaska Tidal Datum Portal tool at https://dggs.alaska.gov/hazards/coastal/ak-tidal-datum-portal.html. Data results from this study are found in the Results and Supplemental tables.
