# Supplementary material for: Current and projected flood exposure for Alaska coastal communities
Source: Sci Rep. 2024 Apr 2;14:7765. doi: 10.1038/s41598-024-58270-w (PMC10987583; doi:10.1038/s41598-024-58270-w)
Supplement: Supplementary file 1 — Supplementary Information. [file 41598_2024_58270_MOESM1_ESM.docx]

# **Flood History, Exposure, and Sea Level Rise Projections Show Substantial Risk for Alaska Coastal Communities**

**S1 Elevation data**

Table 1 lists the DEMs used in this study. All publicly available DEMs are referenced to NAVD 88 on download, although the geoid model and unit may vary. Check metadata to verify the vertical datum, geoid, and units. Following are notes about three primary DEM data sources:

- Topobathymetric lidar data from OCM Partners^[1]^ comes in many formats and datums, including a vertical datum using experimental GEOID18x. A converter from GEOID18x is found at <https://beta.ngs.noaa.gov/GEOID/xGEOID18/computation.shtml>.
- Lidar DTMs from Quantum Spatial^[2]^ and in Deering have vertical datum units in feet. All other datasets are meters.
- When downloading 2015/2016 DSMs from <https://elevation.alaska.gov>, be aware that vertical adjustments have already been applied to community-specific datasets, described by Overbeck et al.^[3]^ Continuous flight-line DSMs described by Overbeck et al.^[4]^ do not have vertical adjustments. At the time this report is written, Deering and Kotzebue do not have community-specific DSMs available for download (only continuous flight-line DSMs), but Glenn et al.^[5]^ compute the necessary vertical adjustment.

Table 1. DEM information for each study site

, including download link, data collection report, GSD, and vertical error at 95% confidence (ZE).

| **Community** | **Elevation Data Source** | **Year** | **Type** | **GSD (m)** | **ZE (m)** |
| --- | --- | --- | --- | --- | --- |
| Alakanuk^a^ | USGS^[6]^ | 2016 | DTM | 1.00 | 0.086 |
| Brevig Mission^a^ | Overbeck et al.^[3]^ | 2015 | DSM | 0.20 | 0.113 |
| Brevig Mission^b^ | OCM Partners^[1]^ | 2019 | DTM | 0.45 | 0.196 |
| Chefornak^a^ | Overbeck et al.^[3]^ | 2015 | DSM | 0.20 | 0.075 |
| Chevak^a^ | USGS^[6]^ | 2016 | DTM | 1.00 | 0.086 |
| Clark’s Point^c^ | DCRA | 2003 | DTM | 1.00 | 0.258 |
| Ekuk^c^ | DCRA | 2003 | DTM | 1.00 | 0.258 |
| Elim^a^ | Overbeck et al.^[3]^ | 2015 | DSM | 0.20 | 0.103 |
| Emmonak^a^ | USGS^[6]^ | 2016 | DTM | 1.00 | 0.086 |
| Golovin^a^ | Southerland and Kinsman^[7]^ | 2013 | DTM | 0.50 | 0.107 |
| Goodnews Bay | Unpublished data from Glenn et al.^[5]^ | 2016 | DSM | 0.20 | 0.211 |
| Hooper Bay^a^ | USGS^[6]^ | 2016 | DTM | 1.00 | 0.086 |
| Kaktovik^a^ | Quantum Spatial^[2]^ | 2018 | DTM | 0.45 | 0.043 |
| Kipnuk | Unpublished data from DGGS | 2021 | DTM | 0.50 | 0.100* |
| Kongiganak^a^ | Overbeck et al.^[3]^ | 2015 | DSM | 0.20 | 0.068 |
| Kotlik^a^ | Herbst and Daanen^[8]^ | 2019 | DTM | 0.50 | 0.082 |
| Kotzebue^a^ | Glenn et al.^[5]^ | 2016 | DSM | 0.20 | 0.320 |
| Koyuk^a^ | Overbeck et al.^[3]^ | 2015 | DSM | 0.20 | 0.125 |
| Kwigillingok | Unpublished data from DGGS | 2021 | DTM | 0.50 | 0.100* |
| Newtok^a^ | USGS^[6]^ | 2016 | DTM | 1.00 | 0.086 |
| Nome^a^ | Overbeck et al.^[3]^ | 2015 | DSM | 0.18 | 0.090 |
| Nome^b^ | OCM Partners^[1]^ | 2019 | DTM | 0.45 | 0.196 |
| Nunam Iqua^a^ | Overbeck et al.^[3]^ | 2015 | DSM | 0.20 | 0.071 |
| Platinum^c^ | DCRA | 2005 | DTM | 1.00 | 0.420* |
| Point Hope^a^ | Quantum Spatial^[2]^ | 2019 | DTM | 0.45 | 0.043 |
| Quinhagak | Unpublished data from Glenn et al.^[5]^ | 2016 | DSM | 0.20 | 0.284 |
| Saint Michael^a^ | Overbeck et al.^[3]^ | 2015 | DSM | 0.20 | 0.033 |
| Scammon Bay^a^ | USGS^[6]^ | 2016 | DTM | 1.00 | 0.086 |
| Shaktoolik^a^ | Overbeck et al.^[3]^ | 2015 | DSM | 0.09 | 0.117 |
| Shishmaref^a^ | AeroMap U.S.^[9]^ | 2004 | DTM | 1.00 | 0.300 |
| Stebbins^a^ | Overbeck et al.^[3]^ | 2015 | DSM | 0.20 | 0.068 |
| Teller^a^ | Overbeck et al.^[3]^ | 2015 | DSM | 0.20 | 0.127 |
| Teller^b^ | OCM Partners^[1]^ | 2019 | DTM | 0.45 | 0.196 |
| Togiak^c^ | DCRA | 2003 | DTM | 1.00 | 0.420 |
| Toksook Bay^a^ | Overbeck et al.^[3]^ | 2015 | DSM | 0.20 | 0.106 |
| Tuntutuliak^a^ | Overbeck et al.^[3]^ Shift of +0.295 m (n = 37). | 2015 | DSM | 0.20 | 0.189 |
| Tununak^a^ | Overbeck et al.^[3]^ | 2015 | DSM | 0.20 | 0.070 |
| Unalakleet^b^ | OCM Partners^[1]^ | 2019 | DTM | 1.00 | 0.196 |
| Wainwright^a^ | Quantum Spatial^[2]^ | 2018 | DTM | 0.45 | 0.043 |
| Wales^a^ | Overbeck et al.^[3]^ | 2015 | DSM | 0.20 | 0.063 |
| Deering^a^ | Metadata provided, no report available. GNSS survey determined error (n = 10). | 2015 | DTM | 0.30 | 0.032 |
| Diomede^b^ | OCM Partners^[1]^ | 2021 | DTM | 0.09 | 0.100* |
| Kivalina^a^ | Metadata provided, no report available | 2016 | DSM | 0.20 | 0.320* |
| Mekoryuk^a^ | USGS^[6]^ | 2016 | DTM | 1.00 | 0.086 |
| Nelson Lagoon | Unpublished data from Bogardus^[10]^ | 2019 | DSM | 0.15 | 0.170 |
| Nightmute^a^ | Overbeck et al.^[3]^ | 2015 | DSM | 0.17 | 0.040 |
| Saint Paul^a^ | Buzard et al.^[11]^ | 2021 | DSM | 0.06 | 0.028 |
| Utqiaġvik^a^ | Quantum Spatial^[2]^ | 2018 | DTM | 0.45 | 0.043 |
| Umkumiut^a^ | Overbeck et al.^[4]^ | 2015 | DSM | 0.20 | 0.106* |

^a^Available at <https://elevation.alaska.gov>

^b^Available at <https://coast.noaa.gov/dataviewer>

^c^Available upon request at <https://www.commerce.alaska.gov/web/dcra/>

*Error value was not published nor able to be evaluated. The value is conservatively estimated based on the typical error for the data collection method.

DEMS were not widely available in southwest Alaska. For Clark’s Point, Ekuk, Platinum, and Togiak, contours are used from Community Profile Maps available from the Division of Community and Regional Affairs website at <https://www.commerce.alaska.gov/web/dcra/>. These are converted to NAVD 88 (geoid12b). Accuracy was computed for all but Platinum through global navigation satellite system (GNSS) surveys conducted by the authors.

To determine DEM suitability, all DEM sources are compared to lidar: contour-derived DTM, crewed aircraft-based structure-from-motion DSM, and uncrewed aerial vehicle (UAV) structure-from-motion DSM (Table 2). Alakanuk is used as a test location because all DEM types are available. For DSMs, the test is also run with a 1 m buffer polygon. This buffer is anticipated to improve DSM comparisons by extending the computation boundary farther from sources of error near the structure. The contour DTM and UAV DSM had the greatest accuracy and precision. Applying the 1 m buffer improved the crewed aircraft DSM offsets and precision. The buffer decreased accuracy of the UAV DSM but increased precision. Based on these results, the 1 m buffer method is applied for DSMs to increase overall accuracy and precision. All sources are suitable to substitute the lidar DTM if needed.

Table 2. Comparison of lidar DTM median elevations to non-lidar DEMs

within infrastructure borders and a 1 m buffer at Alakanuk. Results are the mean offset (DEM - lidar) ± 1 standard deviation. The number of structures changes due to the DEM boundaries. The 1 m buffer improved the standard deviation of DSMs.

|  | Contour DTM | Crewed Aircraft DSM | UAV DSM |
| --- | --- | --- | --- |
| Offset with no buffer | -0.14 ± 0.24 m | 1.50 ± 0.88 m | 0.01 ± 0.43 m |
| Offset with 1 m buffer | N/A | 0.26 ± 0.53 m | -0.18 ± 0.17 m |
| Number of structures | 200 | 200 | 183 |

### **S2 Estimates of Record Floods**

Highest known floods are compiled or estimated using prior analyses and documented flooding, following methods described by Buzard et al.^[12]^. There are three primary sources (listed below). For simplicity, these sources are only given general in-text citations. All other sources are cited normally. The primary sources are:

- hazard mitigation plans (HMPs) available from the Alaska Division of Regional Affairs website (<https://www.commerce.alaska.gov/web/dcra/>). The in-text reference lists the year of the HMP for the community being discussed.
- NOAA Storm Data reports available from the National Climate Data Center website (<https://www.ncdc.noaa.gov>). The in-text reference lists the month and year of the report being discussed.
- USACE investigations. These were publicly available from the USACE Alaska District Floodplain Management website (<https://www.poa.usace.army.mil/>) and summary documents were often copied in HMPs. They are currently not publicly available (March 2023), but the archive was shared with researchers of this study. These include written surveys (flood data questionnaires filled out by residents), site visit reports, and high-water mark survey results. The in-text reference varies and attempts to communicate the contents of the source, as the reader is not anticipated to be able to find these documents online. These sources can be made available upon request.

Flood estimates are made using all available data. Beginning in the 1960s, USACE asked several communities about flood hazards using a Flood Data survey form (Supplementary Figure 1). In the 1990s, USACE visited many communities to identify flood hazards. This resulted in more detailed reports and sometimes temporary benchmark surveys of high water marks (this campaign predates modern GPS technology). A specific goal was to identify the highest known flood, which was assumed to be the 100-year flood. This advised new construction: building first floors were recommended to be built at least 0.3 to 0.6 m above the 100-year flood. This campaign, along with the original written surveys, are often the basis and limit of written flood information available for communities. From the 1980s into the 2000s, the Alaska Division of Regional Affairs developed Community Profile Maps that show the 100-year floodplain determined by USACE for some communities. HMPs also repeat the results of these investigations. The current challenge is to update the flood history beyond the 1990s and convert prior study results to the current tidal and orthometric datum.


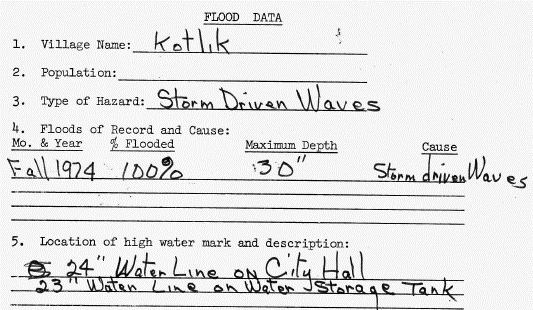


Supplementary Figure 1. Example of a section of a filled out Flood Data survey form for Kotlik.

All elevations in m NAVD 88 use geoid12b unless otherwise specified. Conversion to local MHHW are computed using the Alaska Tidal Datum Portal (November 2022 Update; <https://dggs.alaska.gov/hazards/coastal/ak-tidal-datum-portal.html>). Conversions between non-tidal datums are computed using VDATUM Online (Version 4.6; <https://vdatum.noaa.gov/vdatumweb/>). Uncertainty is reported at a 95% confidence interval.

Alakanuk

The highest known storm surge flood reached 6.5 ± 0.5 ft MHHW (4.49 ± 0.15 m NAVD 88) on November 10, 1974^[13]^. The published datum is 1.8 ft lower than the unpublished datum used in the report. This flood caused major impacts.

Brevig Mission

USACE investigations indicate the highest known flood occurred November 10, 1974. USACE estimated the elevation with a non-GPS survey. DCRA later surveyed the USACE estimate with GPS to be 3.237 m NGVD1929. The conversion from NGVD1929 to NAVD 88 (geoid12b) is + 0.958 m at Brevig Mission, bringing the estimate to 4.195 m NAVD 88. The beach fronting the community has a berm cresting approximately 4.4 m NAVD 88, and reports indicate buildings set behind this berm have never flooded despite being lower than 4.195 m NAVD 88. The berm appears to prevent significant flooding of the landward, lagoon side. Waves did reach a building atop the berm in 1974. The estimated elevation is consistent with reported impacts. The uncertainty of the elevation is not published, so a conservative value of 0.5 m is applied. This was a moderate flood.

Chefornak

The 2014 HMP does not identify the highest known flood. The report quotes a USACE report from 1967, “The village is located on a small rock outcropping and has 3-4 ft of freeboard above the flood stage. There is no record of flooding in the community. However, the surrounding area is extremely low and is subject to frequent flooding.” Chefornak is 8 miles from the coast, up a meandering river. The community relocated to its current location in the 1950s to evade flooding^[14]^. Since 1967, some structures have been built in low-lying areas beyond the rock outcropping. The highest known flood estimated by DGGS reached 3.6 ± 1.3 ft MHHW (3.17 ± 0.40 m NAVD 88) on October 4, 2012 (DGGS, personal communication, April 2023). This is estimated to cause moderate impacts. The estimate comes from a DGGS flood study in progress and is subject to change or be exceeded if a higher event is discovered.

Chevak

USACE and HMPs do not identify the highest known flood. The fish camp area flooded in 1979, 1981, and 1988, but the cause of these floods is not identified (the area is subject to riverine flooding from spring snowmelt). UAF surveyed high water marks of the September 17, 2022 flood, averaging 6.1 ± 1.0 ft MHHW (3.91 ± 0.32 m NAVD 88; n = 39; SD = 0.16 m). Community elders told UAF surveyors that this flood rivaled a record flood from 50 to 60 years prior (1970s to 1980s). Technically this was a minor flood, but a significant amount of subsistence equipment was damaged.

Chignik Bay

The 2019 and 2015 HMPs explain that flooding can occur from storms or high tide and spring melt. USACE investigations in 1992 identified the flood of record occurred in October 1948. The HMP describes other notable events that flooded the airport runway, damaged coastal infrastructure, and flooded homes and other structures. The 2002 Community Profile Map incorporates the USACE survey and shows the elevation of the 1948 flood is 6.0 ft below a nearby tidal datum benchmark “945 8917 I TIDAL.” The benchmark is listed on the map as 15.6 ft MHW, but the NOAA tidal datum benchmark sheet shows 11.91 ft MHW. This brings the flood estimate to 5.91 ft MHW. Uncertainty of the USACE survey is estimated to be 0.5 ft. The October 1948 flood reached 5.1 ± 0.5 ft MHHW (3.79 ± 0.15 m NAVD 88). This was a major flood.

Clark's Point

Clark’s Point is partially built on a spit with a broad back-barrier marsh, similar to neighboring Ekuk. The highest features on the spit are the alongshore crest, the old airstrip, and the cannery. The ground elevation for these features averages 4.1 ± 1.0 ft MHHW (5.26 ± 0.29 m NAVD 88). The 2019 HMP indicates that Clark’s Point occasionally experiences major flooding, impacting tank farms, public and private structures, and the cannery. The May 1985 flood is listed as the highest known event. The October 2005 storm may have caused the most significant damage, but the height is not described by the HMP. The August 2005 Storm Data report describes major flooding in Clark’s point, including flooding of 4 residences and submergence of the runway. USACE flood hazard data from 2000 states, “The worst flood event occurred in November, 1929, with water reaching a depth of 4 ft. Water reached a depth of 3 to 4 ft in the flood of October 1964, 2.5 ft in the flood of August 1980, and about 6 inches at some home sites in the flood of December 1960. A May 1985 storm drove waters 6 ft above normal levels, damaging the clinic’s foundation and flooding truck motors and dry docked boats with open drain ports. The last flood occurred in October 1995 when approximately 1.5 ft of water covered the airstrip. Flooding occurred in November 1949, but flood depths were not reported.” The record flood appears to be November 1929 because it was listed as the deepest and worst by USACE, and listed as deeper than in May 1985, the record indicated by the 2019 HMP. Reports do not list the location where depths are estimated. The airstrip is 4.2 ± 0.8 ft MHHW, similar to the surrounding spit, and had 1.5 ft of water in October 1995, a non-record event. The record is estimated to have exceeded this level (2 ft depth) but not exceeded 4 ft depth, placing the range at 3.0 ± 1.0 ft depth at the airstrip. The November 1929 flood is estimated to have reached 7.2 ± 1.1 ft MHHW (6.20 ± 0.34 m NAVD 88).

USACE lists the 100-year flood elevation as 32 ft MLLW, and MHHW is listed as 19.5 ft above MLLW. The published datum has a 20.67 ft range. This places the 100-year flood estimate between 11.3 to 12.5 ft MHHW (7.45 to 7.82 m NAVD 88). It is not clear how the 100-year flood was originally estimated, but the result appears to be several feet above observed flooding.

Deering

USACE investigations indicate the highest flood in Deering occurred in 1978. A high-water sign was placed on a telephone pole in front of the store, along with a recommended building height sign 1.2 ft above. DGGS surveyed the building height sign in Deering in 2018 (the high-water sign was missing), measuring 11.3 ft MHHW (4.91 m NAVD 88). The high-water sign was 10.1 ft MHHW (4.55 m NAVD 88).

The listed date of the record flood is questionable. A flood of this elevation would inundate the entire spit with at least 1 ft of water. A Bering Sea storm on November 8-10, 1978 did cause flooding in Nome, but no sources, including USACE, describe this storm impacting Deering. Only two storms are described causing this level of flooding in Deering: November 1973 and November 1974. The November 1974 storm caused 2 ft of flooding at the store next to the high-water sign. However, the November 1974 Storm Data report only cites this as causing minor damage in Deering. Delayed reporting from the June 1974 Storm Data report states, “At Deering 17 dwellings were lost” in November 1973. This level of impact is unprecedented, so the 1973 storm is likely the flood of record, not 1978. Based on the high-water sign, the November 10-15, 1973 flood reached 10.1 ± 1.0 ft MHHW (4.55 ± 0.3 m NAVD 88). The uncertainty is estimated to be 1.0 ft.

Diomede

USACE states the highest known flood in Diomede occurred December 29-30, 1977, when a storm traveled from the Bering Sea to the Arctic. USACE collected two written surveys. One survey states the depth of flooding was 15 to 20 ft, but a flood of this height would cause several impacts that were not observed. The other observation says the high-water mark is 10 ft above sea level. Diomede does not have a tidal datum, but reports indicate there is a small tidal range of about 1 ft like nearby Wales. Observations of depth and elevation above sea level are effectively in the same datum (± 0.5 ft). The 1980 Community Profile Map shows the lowest-lying infrastructure at the time were eleven fuel tanks around the water tank. The median ground elevation under structures at this location is 3.62 ± 0.51 m NAVD 88. Using the Wales tidal datum, this is 8.2 ± 1.7 ft MHHW. The tanks were not on a large, raised platform and did not have a walled perimeter like many modern tank farms. Photos of flooded fuel tank farms in villages in the 1970s show that fuel tanks were typically placed on the ground or on relatively short platforms (e.g. Buzard and Overbeck^[15]^). The estimated flood level of 10 ft above sea level would cause approximately 1 to 2 ft water depth at the tank area, enough to float unsecured fuel tanks. The surveys did not observe damage to the fuel tanks, but the tanks were damaged in a 1990 flood.

The 2019 HMP describes a November 21, 1990, storm “… with waves up to 25 feet destroyed several fuel storage facilities. The resultant loss of critically needed petroleum products along with other equipment, required the declaration of disaster.” USACE provided a print article from Alaska Daily News on November 25, 1990, that explains this storm occurred over the previous weekend and culminated on Monday the 18^th^. A second, smaller storm occurred Thursday, the 22^nd^. Since fuel tanks were damaged, flooding likely exceeded the 1977 event. The aerial image used in the 1996 Community Profile Map was collected in 1994 and shows the two Native Store bulk oil tanks seen in the 1980 map are no longer there, and new bulk fuel tanks have been placed on higher ground to the south (near the areas labeled “potential rockslide area,” “area of active erosion,” and “community meat racks”). The nine school fuel tanks are still there but are no longer labeled, and a new installation of school fuel tanks is to the north.

The two main conclusions drawn from these details are (1) the 1990 storm was more damaging to the fuel tank area than in 1977, and (2) water must have reached the fuel tank area and had enough depth, velocity, or wave energy to damage the tanks. A depth of 2 ± 1 ft at the tank area appears to be consistent with estimates of water levels and would remain low enough to not flood nearby structures. This brings the total depth of the November, 18, 1990 flood to 10.2 ± 2.0 ft MHHW (4.23 ± 0.61 m NAVD 88). Uncertainty is the RSS of the depth estimate, tidal range, and ground elevation uncertainty.

Ekuk

Ekuk is built on a broad, low-lying spit that floods due to storm surge and spring runoff. The 2019 HMP describes a major flood in fall 2018 that effectively submerged the spit and damaged several cabins. The HMP indicates community-wide flooding has happened multiple times. A written survey collected by USACE in 1973 identifies three more events: November 1929, 1966, and 1969. The 1969 flood appears to be the listed record at the time, flooding about 2 ft deep at the cannery area. The cannery is a large area around a slightly elevated point at the end of the spit. We surveyed this area to be between 3.3 and 6.5 ± 0.1 ft MHHW (5.02 and 6.00 ± 0.08 m NAVD 88). A flood elevation of 5.3 ± 1.0 ft MHHW would inundate this area with up to 2 ft of water. Structures are built in a row along the high ridge of the spit, averaging 4.6 ± 2.1 ft MHHW (5.42 ± 0.66 m NAVD 88). Flooding of 5.3 ft MHHW would inundate the area of most structures, effectively submerging the spit as has been observed. The 1969 flood is estimated to have reached 5.3 ± 1.0 ft MHHW (5.62 ± 0.30 m NAVD 88). This was a major flood. Although the November 1929 flood impacts are not described for Ekuk, that was the record flood event for Clark’s Point 1 mile north and exceeded this estimate by 1.9 ft (0.58 m). We use the Clark’s Point estimate for Ekuk for this study.

Elim

USACE investigations found the highest known flood occurred October 28, 1945. USACE estimated the elevation with a non-GPS survey in 1997. DCRA later surveyed the USACE estimate with GPS to be 5.47 m NGVD 1929. This is 6.39 m NAVD 88 (15.7 ft MHHW). The uncertainty is estimated to be 1 ft (0.3 m). Storm impacts are not described, but USACE states the elevation was similar to storms in November 1974 and in 1917 (and/or 1913). The November 1974 flood was 2 ft below five residences. The 1945 flood is estimated to have moderate impacts.

Golovin

The highest known flood in Golovin reached 12.0 ± 2.0 ft MHHW (5.25 ± 0.61 m NAVD 88) on October 28, 1945^[16]^. This was a major flood.

Goodnews Bay

The highest known flood in Goodnews Bay reached 11.2 ± 0.5 ft MHHW (5.5 ± 0.15 m NAVD 88) on November 11, 2011^[17]^. This was a major flood.

Hooper Bay

The highest known flood in Hooper Bay reached 7.7 ± 1.4 ft MHHW (4.41 ± 0.43 m NAVD 88) on September 22, 2005^[15]^. This was a major flood.

Kaktovik

Storm surge flooding has not reached structures in the community. The 1964 flood was the highest documented event by USACE in a 1993 survey. The 1964 flood reached 5.77 ft MSL, which is 5.45 ft MHHW (2.86 m NAVD 88). The uncertainty is estimated to be 1.0 ft (0.3 m). This was a minor flood. No reports described the month or cause.

Kipnuk

The highest known flood in Kipnuk reached 4.5 ± 0.2 ft MHHW (3.73 ± 0.06 m NAVD 88) on October 28, 2016 (personal communication, DGGS, April 2023). The estimate comes from a DGGS flood study in progress and is subject to change or be exceeded if a higher event is discovered.

Kivalina

According to the 2007 HMP, Kivalina was established on an island in 1905 and has experienced three storms that “…overtopped portions of the island…” Currently the lowest portion of the island is just northwest of the runway and water would have to exceed 8.6 ± 1.0 ft MHHW to overtop it. Storms have caused considerable erosion, but there are no reports describing flooding of structures. A letter to USACE from DCRA in December 1973 summarizes flood risk after visiting Kivalina, “There is no evidence that flooding has occurred within the village. Conversations with village residents indicated that occasionally water on the lagoon side has been within a few feet of flooding the village; however, actual flooding has not occurred within the last thirty years… Buildings did not possess high water marks nor was there evidence of drift materials.” Buildings are built a few feet above ground. Ocean-side structures are built on ground averaging 11.1 ft MHHW (SD = 1.2 ft; n = 40), with the lowest at 8.9 ± 1.0 ft MHHW. Lagoon-side structures are built on ground averaging 7.3 ft MHHW (SD = 1.5, n = 41) with the lowest at 6.0 ± 1.0 ft MHHW. Lagoon-side structures are protected from open ocean waves.

The 2015 HMP and USACE investigations state that the highest observed flood occurred in September 1970 and was approximately a 50-year flood. Chapman et al.^[18]^ modeled a 50-year storm surge reaching 6.0 ft MHHW (SD = 0.9 ft). This was one of the events that overtopped a portion of the spit. This places the minimum runup estimate at 8.6 ± 1.0 ft MHHW. Aerial imagery from 1978 shows structures were in the same location as they are today. To be high enough to overtop portions of the spit but low enough to not flood structures on the ocean side, the maximum wave setup value must be below 8.9 ± 1.0 ft MHHW. The September 1970 flood reached 6.0 ± 1.8 ft MHHW (2.90 ± 0.55 m NAVD 88) with wave setup reaching 8.8 ± 1.2 ft MHHW (3.75 ± 0.37 m NAVD 88). Although storms cause erosion, the elevation below infrastructure designates this as a minor flood. We rely on the modeled value for this event surge because the model is supported by the nearby water level sensor at Red Dog Dock. The modeled value is near the ground elevation of lagoon-side structures, consistent with observations.

Record flood date and elevation estimates: The reported month and elevation vary depending on the source. The 1970 flood is first described in a written survey in 1970 and listed as occurring in Sept., flooding “20-30%” of an unspecified area, having a maximum depth of “2 ft along the beach,” and reaching a maximum elevation of 15 ft MLLW (14 ft MHHW). Chapman et al.^[18]^ did not model a September 1970 event, but identified the November 1970 storm as being the third highest flood in the study period. The November 1970 Storm Data report describes flooding in Kotzebue, but not Kivalina. The September event is not mentioned in Storm Data reports. There was a September 1970 storm that caused flooding in Brevig Mission^[19]^. This is the only source other than the initial survey to describe a September 1970 flood. We list this as the September 1970 flood given that a storm did occur at that time and the source material listed it as September but acknowledge this could have occurred in November or there could have been two events. Impact descriptions show the flood did not reach 15 ft MLLW. Also, Wise et al.^[19]^ lists a September 1976 flood that flooded 20-30% of the community with a surge of 15 ft MLLW. There is no other record of a September 1976 flood, and the description is identical to 1970, so the entry may have meant to be for 1970.

Incorrect models and interpretations: Buzard et al.^[12]^ discuss how Kivalina has a history of inaccurate models of the 100-year flood. The Chapman et al.^[18]^ model is regarded as the most accurate, and modeled the 100-year flood to be 6.9 ft MHHW, not including wave setup and runup. In 1998, USACE published a Community Improvement Feasibility Report for Kivalina that estimated the 100-year flood is 9.7 ft MHHW including wave setup. The 2021 HMP and other sources claim that a 100-year event would flood the entire spit. However, the modeled 100-year flood is below the elevation of most of the townsite. The lagoon side is as low as 6 ft MHHW and would experience approximately 1 ft of flooding in a 100-year event.

Kongiganak

Kongiganak is built on a plateau about 15 ft (4.5 m) above the surrounding lowlands. The community is 5.5 miles upstream from the coast, but storm surge still reaches this area. The 2015 and 2021 HMPs indicate flooding occurs from storms and high river levels during spring. Flooding has never reached structures in the community but has damaged boardwalks, as occurred from a storm on November 2000. USACE floodplain management estimates the record flood elevation is 20.7 ft MLLW. This is 10.3 ft MHHW (6.03 m NAVD 88). The plateau is about 10 ft (3 m) above the 100-year estimated flood. A flood of this elevation would cause minor flooding, but damage subsistence equipment left at low elevation. Although the record date is unknown, the elevation appears to be consistent with accounts of minor flooding. An uncertainty of 2.0 ft (0.61 m) is given because a high-water mark is not identified. It is unclear if this reflects a storm surge event or a high river level event.

Kotlik

The highest known flood in Kotlik reached 7.7 ± 0.4 ft MHHW (4.25 ± 0.12 m NAVD 88) on November 10, 1974^[20]^. This was a major flood.

Kotzebue

The Kotzebue 2014 and 2019 HMPs provide a list of historical flood events. However, the list is the storm surge simulation results by Chapman et al.^[18]^, which are not validated and may not have occurred due to sea ice and other factors limiting modeling efforts. The HMPs list the August 25, 2012, storm as the record, reaching 10 ft “above normal tide level.” The Kotzebue great diurnal range is only 0.71 ft. The source for this estimated elevation comes from the August 2012 Storm Data report that states the flood nearly inundated the airstrip, which is 10 ft above the lagoon. The minimum elevation of the airstrip centerline is 4.7 ± 1.0 ft MHHW (2.65 ± 0.32 m NAVD 88). The storm surge level did not exceed this elevation. Imagery collected by NWS shows flooding of the windsock on the northeast section of the runway, where ground is 2.5 ± 1.0 ft MHHW (2.0 ± 0.32 m NAVD 88). The storm surge exceeded this by at least 1 ft. A high-water line seen on the tire of a car in a parking lot of an apartment complex shows water was 0.75 ± 0.25 ft above ground, bringing the total to 3.6 ± 1.1 ft MHHW (2.33 ± 0.33 m NAVD 88). This is the clearest high-water line and fits within the minimum and maximum surge elevations. The August 2012 Storm Data report states the seawall remained above water. The seawall averages 6.0 ± 1.6 ft MHHW (3.05 ± 0.48 m NAVD 88). The August 2012 storm surge is estimated to have reached 3.6 ± 1.1 ft MHHW (2.33 ± 0.33 m NAVD 88), not 10 ft as listed in the HMPs. On the exposed coast where wave setup occurs, water reached near 6.0 ± 1.6 ft MHHW (3.05 ± 0.48 m NAVD 88).

The seawall, built in 2011, impedes wave setup and runup. Previously, the highest known flood in Kotzebue occurred August 26, 1990^[21]^. This storm flooded four coastal homes that were built at ground level with approximately the same elevation as the seawall. Water may have exceeded the level of the 2012 event but was unimpeded because the seawall was not constructed. Since the 2012 event rivaled 1990 and reflects the influence of the seawall, this is used as the record flood. Other significant floods that rivaled these events occurred in September 1986, 1964, and August 1955. None of these observed storm events are listed in the HMPs.

Koyuk

The Koyuk 2014 HMP states the highest flood in memory reached 14 to 15 ft MLLW (11 to 12 ft MHHW) on November 10, 1974, citing the high-water mark survey conducted by USACE in the 1990s. USACE notes the highest flood is technically the October 5, 1913, flood that left a driftwood line. Driftwood lines are visible in aerial imagery collected in 2015. A 250-ft long driftwood line at the second vegetated valley approximately 600 m east of town averages 13.1 ft MHHW (SD = 0.2 ft). This is approximately 1 to 2 ft above the 1974 flood, a result consistent with findings of nearby Golovin^[16]^. An uncertainty of 2 ft is used to conservatively account for the sources of error around using driftwood line. The October 5, 1913, flood is estimated to have reached 13.1 ± 2.0 ft MHHW (5.74 ± 0.61 m NAVD 88). Impacts of this event are not described. A written survey collected by USACE in 1986 describes a fall 1917 storm that flooded 2 houses of 23. This would have been a major event, so the record event exceeding it was also a major flood.

Mekoryuk

The 2015 HMP states Mekoryuk does not experience flooding. USACE estimated the November 10, 1974 storm surge reached 5.9 ft MHHW (3.9 m NAVD 88) based on observations of flooding at the beach. Uncertainty is estimated to be 2.0 ft given there is no specific high-water mark. This does not qualify as minor flooding.

Napakiak

The highest known storm surge flood reached 5.22 ± 0.34 m NAVD 88 on August 17, 1990^[22]^. This was a major flood. Napakiak does not have a tidal datum but is about midway between tidal datums of Lomavik (MHHW is 2.973 m NAVD 88) and Bethel (MHHW is 3.258 m NAVD 88). Both are tied to NAVD 88. Using the average datum conversion (3.116 m), the flood is estimated to be 6.9 ± 1.5 ft MHHW. Uncertainty is the RSS of the original estimate and half the range of the datum conversion.

Newtok

Newtok does not have a published record flood level. Newtok is built on a plateau that is highest near the shoreline with gradually lower elevation inland. The highest point is about 10 ft MHHW (5.1 m NAVD 88) and the lowest area where structures are built is around 4 ft MHHW (3.3 m NAVD 88).

The 2015 HMP describes some flood impacts. The highest flood described occurred September 22-23, 2005. The village was “completely enclosed” by water, which reached underneath several homes and floated boardwalks. About 2 ft of water above the lowest area is enough to cause the temporary island as observed in 2005. This would be 6 ft MHHW.

USACE collected several written surveys of flood history. All surveys state no homes have been flooded and all but one state no flood has ever occurred. One survey in 1980 explains that the worst known flood occurred in October 1979 and reached 10 to 12 feet above high tide. It came close to two homes but flooded neither. Storm Data records do not show an October 1979 storm in this area, but two notable storms occurred in November 1979 that caused major flood impacts in southwest Alaska. A flood of 10 ft MHHW would flood the entire community. This contradicts all sources, so the reported elevation is likely overestimated. Relatively low flooding can reach underneath some structures, and the structure elevation above ground level is not known, so the flood cannot be accurately estimated.

The highest known flood in Newtok reached 6 ± 2 ft MHHW (3.9 ± 0.6 m NAVD 88) on September 22-23, 2005. Uncertainty is estimated to be 2 ft because there is not a clear high-water mark to measure. This was a moderate flood.

Nightmute

Nightmute is about 10 miles upstream and rarely experiences storm surge flooding. USACE investigations identified the November 2011 storm as causing the highest flood recorded in Nightmute. In August 2012, USACE surveyed three high-water marks in the NAVD 88 datum but did not specify a geoid. The two ground-level high-water marks identified by USACE were identified in aerial imagery and measured 1.9 ± 0.6 ft MHHW (2.90 ± 0.18 m NAVD 88) using the DSM and Toksook Bay’s tidal datum. This was a minor flood.

Nome

The highest known flood for Nome occurred on October 5, 1913. The flood insurance study conducted by USACE for FEMA found reports at the time stating the storm reached 14 ft above “ordinary high tide.” Based on water level measurements from 1992 to 2021, RSLR at Nome is 1.28 ± 0.82 ft per century (NOAA CO-OPS station 9468756). If this was the rate since 1913 and the storm reached 14 ft MHHW, then the storm reached the equivalent of 12.5 ft MHHW today. This is 2 ft above the November 1974 flood in Nome, according to USACE estimates listed in the 2008 HMP. The RSS of the source uncertainty (2 ft) and the RSLR uncertainty is 2.2 ft. The October 5, 1913, flood is estimated to have reached 12.5 ± 2.2 ft MHHW (5.06 ± 0.67 m NAVD 88). This was a major flood.

Nunam Iqua

The highest known flood reached 8.5 ± 1.0 ft MHHW (4.57 ± 0.30 m NAVD 88) on November 5, 2013^[23]^. This was a major flood.

Platinum

Platinum is a small community and one of the few without a HMP. In 1994, USACE surveyed the record flood level relative to a temporary benchmark of the concrete floor of the powerhouse set to 100 ft. The November 9, 1979 flood caused 9 inches of water above the first floor of a house surveyed to 101.4 ft, making the flood level 102.15 ft. On August 17, 1988, water flowed underneath an old house with a surveyed elevation of 98.8 ft. The water depth is not specified, but a survey photo shows the first floor is approximately 2 ft above ground, so the flood likely reached between 99.8 and 101.8 ft to flow underneath the home without reaching the first floor. The 2004 Community Profile Map has contours in ft NAVD 88 (geoid99). The survey photo of the powerhouse shows the concrete floor is approximately ground level. The powerhouse is on the 6 ft contour, so subtracting 94 ft from the temporary benchmark survey converts values to NAVD 88 (geoid99). This adjusts the 1979 flood to 8.15 ft and the 1989 flood to 5.8 to 7.8 ft NAVD 88 (geoid99). Both surveyed houses are between the 4 and 6 ft contour, so flooding of approximately 2 ft above ground is consistent with observed high-water levels. Geoid99 is 9.13 ft above geoid12b, so the elevations are again adjusted to be 17.3 ft and 14.9 to 16.9 ft NAVD 88 (geoid12b). The final estimates for the 1979 and 1989 storms are 8.6 ± 1.0 ft MHHW (5.27 m ± 0.30 NAVD 88) and 7.2 ± 1.4 ft MHHW (4.84 ± 0.43 m NAVD 88), respectively. The record event (1979) is approximately the 8 ft contour on the 2004 Community Profile Map. The uncertainty of the 1979 storm is estimated to be 1.0 ft given the relatively precise survey, but also considering limitations and datum conversions. The uncertainty for 1989 is the RSS of this initial uncertainty (1 ft) and 1 ft uncertainty of the water depth.

Point Hope

The highest known flood occurred October 13, 1893^[24]^. A resident that weathered the storm explained, “… one to three feet of water covered nearly all the western portion of the spit”^[24]^. The community was built in the northwest area at the time. Waves destroyed the door of a chapel that was approximately 16 ft above sea level and 250 ft inland. The structure elevation is approximated, not surveyed. The current tidal range is 0.67 ft, seemingly unchanged given the description by Kindle^[24]^ that, “the tide at Point Hope is probably considerably less than two feet…” The 2017 Point Hope Comprehensive Plan explains, “The entire village flooded in 1893, when a 10-foot storm surge covered the spit.” The spit is a series of dunes. The previous community location west of the current runway is still evident in aerial imagery and DEMs. Water level of 10 ft would inundate the beach and reach near the vegetation line. Where structures were built, the dunes peak at 10.8 ± 0.6 ft MHHW. Water reaching 11.8 ft MHHW would cover the dune peaks with 1 ft of water minimum, consistent with observations. The October 13, 1893, flood is estimated to have reached 11.8 ± 0.8 ft MHHW (4.74 ± 0.25 m NAVD 88). Uncertainty is the RSS of the dune peak elevation uncertainty, water depth estimate uncertainty (0.5 ft), and half the tidal range.

Quinhagak

The Quinhagak 2020 HMP states the highest known storm surge floods occurred August 17, 1989 and November 11, 2011. A higher ice-jam flood occurred in 1978. The 1989 event damaged residences. The 2011 storm flooded low-lying areas with fish drying racks but no other cited damage. The 2012 HMP states 2011 was not as high as 1974, but there is no other mention of 1974. The September 2005 storm also flooded all low-lying areas (2012 HMP).

Quinhagak is built on a plateau overlooking floodplain terraces of the Kanektok River^[25]^. Fish drying racks and subsistence equipment are found in the floodplain. Aerial imagery from 1982 shows residences were built on the same plateau as today. The lowest residences are built where the ground is between 7.5 and 10.0 ft MHHW. It is unclear how specifically the 1989 storm damaged residences or how deep the water was. The August 17, 1989 flood estimate is the halfway mark of the low-lying residence ground elevation, 8.8 ± 1.3 ft MHHW (5.66 ± 0.40 m NAVD 88). This caused minor to moderate flooding.

The flood of record was an ice-jam flood in 1978. In 1994, USACE estimated the water reached 9.0 ft “above sea level.” Community planning documents assume this is in reference to MSL, but USACE did not have a tidal datum and may have been referring to the high tide line (MHW or MHHW), river level, or highest observed water while visiting the city. Quinhagak has a great diurnal range of 11.9 ft. MSL is 6.5 ft below MHHW. It is unlikely USACE was referring to MSL, as the record flood would only be 2.5 ft MHHW and have no impact on the community. Reference to MHW or MHHW would make the flood of record 6.8 to 9.0 ft MHHW (5.0 to 5.8 m NAVD 88). This flood is listed as being higher than in 1989, so the elevation is closer to 9.0 ft MHHW than 6.8 ft MHHW.

Saint Michael

Saint Michael is built on a rocky plateau above the floodplain. In 1993, USACE estimated the record flood elevation was 16.9 ft MLLW (14.2 ft MHHW, 5.79 m NAVD 88) and occurred between 1959 and 1988. USACE estimates the 100-year flood is 20.0 ft MLLW (17.3 ft MHHW) but do not claim such an event was observed. The highest known flood date is never disclosed in USACE or HMP reports, but the water level is said to have reached the footing but not the first floor of the Yutana Barge Lines building. The building was constructed in 1900 and had never flooded before. The ground elevation is 11.7 ± 0.1 ft MHHW (5.0 ± 0.03 m NAVD 88), and the first floor appears to be 2.5 ft above the ground based on photographs. A water level of 1.25 ± 0.75 ft above ground level would reach the footing without flooding the first floor. The record storm surge flood is estimated to have reached 13.0 ± 0.8 ft MHHW (5.41 ± 0.24 m NAVD 88). There are four floods described as causing minor impacts: 1964, 1970, 1971, and November 1974. Given the 1974 storm is the record for neighboring communities, we estimate this was also the record for Saint Michael. This was a minor flood. The record flood elevation estimated by USACE in 1993 is slightly overestimated.

Saint Paul

The highest known flood in Saint Paul reached 12 ± 2 ft MHHW (3.8 ± 0.6 m NAVD 88) on December 25, 1966. This estimate comes from a flood history study currently being conducted by the Arctic Coastal Geoscience Lab. Uncertainty is estimated to be 2 ft until the study is published. Water reached the foundation of several homes and was ankle-deep at the gas station area at the time. This storm caused moderate flood impacts.

Scammon Bay

According to the 2013 HMP, the record flood event was caused by rainfall in September 1981. The record storm surge flood event was in August 1976 and flooded the airstrip, sewage lagoon, and two homes, with a cited depth of 6 ft (no datum or location provided). The airstrip has been expanded and elevated since then, but the current airstrip is below 6.3 ft MHHW. The current lagoon berm has low points between 6.3 to 8.0 ft MHHW. Structures are mostly built on a plateau above 10 ft MHHW that has never flooded. The flood must have reached at least 6 ft MHHW to be 6 ft deep, but not exceed 10 ft MHHW. This places the estimate at 8 ± 2 ft MHHW. This depth would inundate the airstrip and sewage lagoon, consistent with observations. The lowest-lying structures are on ground between 2 and 6 ft MHHW, so a flood of 8 ft MHHW could inundate them if they are not high above the ground. The August 1976 flood reached 8 ± 2 ft MHHW (4.5 ± 0.6 m NAVD 88). This caused major flood impacts.

Shaktoolik

Shaktoolik experiences wave setup and runup that reaches higher than the surge on the ocean-facing west side of the community^[26]^. The current community is built along a spit cresting at the main road that averages 17.6 ± 1.0 ft MHHW (7.22 ± 0.30 m NAVD 88). According to the 2015 HMP, the village was originally 6 miles up the Shaktoolik river. In 1933, the village moved to an area of the spit 2 km southeast of the current townsite where the berm crests at approximately 21 ft MHHW. On October 2, 1960, this area saw major flooding with total water level reaching nearly 24 ft MHHW^[27]^. In 1967, the village moved again to avoid flooding, this time to the current location. Although the current site is lower in elevation, USACE^[27]^ explains, “Coastal flooding of this magnitude has not been seen at the current community site.” Kinsman and DeRaps^[26]^ also observed this phenomenon; the November 2011 storm runup reached up to 23.4 ft MHHW at the old site, but only an average of 16.6 ± 0.6 ft MHHW at the current site. Kinsman and DeRaps^[26]^ suggest this 6 ft difference may be due to nearshore water depths varying between the two locations. At neighboring Unalakleet, Erickson et al.^[28]^ observe a similar phenomenon and attribute it to an alongshore bar or shoal offshore of the community that is not present farther down from the spit. While the cause is not identified at Shaktoolik, Kinsman and DeRaps^[26]^ suggest flood levels at the old town site should not be assumed to have been the same level at the current site.

For this study, the November 2011 storm is used to estimate the record event observed at the current community site. Water reached a similar elevation in November 2013, November 2009, and September 2005. Some of these events may have slightly exceeded 2011, but the greatest documentation exists for 2011. The record flood level is 16.6 ± 0.6 ft MHHW (6.91 ± 0.18 m NAVD 88). The still water level is 8.9 ± 0.9 ft MHHW (4.57 ± 0.29 m NAVD 88)^[26]^. This caused major impacts in the current community.

Shishmaref

Shishmaref is built on an elevated spit where most structures are above the floodplain, but significant erosion undercuts structures. Corresponding between USACE surveyors and the community in the 1970s determined that the November 10, 1973 storm was the highest flood in the memory of elders, and only rivaled by a flood experienced by their great grandparents. Water submerged both ends of the airport. Water reached up to the southern edge of the community building and the AVEC Power Plant. The village structures were not flooded, but substantial damage occurred to vehicles, subsistence equipment, and food stores in low-lying areas or on the bluff edge where considerable erosion occurred. USACE estimated the flood reached 8 ft MSL on the ocean side and 6 ft on the lagoon side, and these values are used for the FIRM. These values would cause the described flooding at the runway at the time and near the power plant. The record flood of November 9-10, 1973 is estimated to have reached 7.5 ± 1.0 ft MHHW (3.35 ± 0.30 m NAVD 88) on the exposed coastline and 5.5 ± 1.0 ft MHHW (2.74 ± 0.30 m NAVD 88) on the sheltered side. Uncertainty is the RSS of the 1 ft implied uncertainty from single-figure USACE estimate. This caused moderate flood impacts.

Stebbins

The highest known flood in Stebbins reached 14.2 ± 1.0 ft MHHW (5.79 ± 0.30 m NAVD 88) on October 2-3, 1960 (personal communication, DGGS, March 2023). Four to five of the thirty homes were flooded with 1 ft of water, causing major flood impacts. The estimate comes from a DGGS flood study in progress and is subject to change or be exceeded if a higher event is discovered.

Inaccurate model: USACE^[29]^ calculated the flood frequency curve based on 7 floods in 30 years and extrapolated a 100-year flood elevation prediction of 17.3 ft MHHW, 3.1 ft above the highest known flood. Records to-date indicate no flood has reached higher since 1960. USACE^[29]^ interviewed a resident that recalled only one event in the 1910s or 1920s that may have reached or exceeded the 1960 flood elevation. Using the same method as USACE^[29]^, this extended record places the 1960 flood as a 50- or 100-year event. In other words, the estimated 100-year flood elevation would be closer to 14.2 ft than 17.3 ft MHHW using this method.

Teller

The highest known flood in Teller occurred October 5, 1913. USACE flood surveys have no specific descriptions other than water reached 2 ft above the 1974 flood. The 1974 flood reached 29 inches above the ground at the Post Office. The Post Office location is identified in the 1980 Community Profile Map and is in the same location today. The 2022 HWM survey measured the Post Office ground at 5.0 ft MHHW, making the 1974 flood 7.4 ft MHHW. Given the limited information about the 1913 event in Teller, an uncertainty of 2 ft is used. The October 5, 1913 flood is estimated to have reached 9.4 ± 2.0 ft MHHW (4.0 ± 0.6 m NAVD 88). This caused major flood impacts.

Togiak

Togiak has a FEMA FIRM. The 2010 Flood Insurance Study explains that people began gradually settling the current townsite as early as the 1880s. The October 1964 flood is regarded as the record event, and afterward many residents left to found Twin Hills on high ground across the bay and upriver. Several written surveys collected by USACE in the 1980s state that the entire village was flooded with 2 to 4 ft of water. Reports indicate all residences and most other structures were flooded, and some houses floated off their foundations. Several other floods have occurred, although details vary by source. The 2019 HMP states major floods also occurred in 1979, 1980, and 1982. Written surveys indicate minor floods occurred in 1981, 1982, and 1983. One survey identified a flood in September 1965 that flooded 2 to 3 ft, just lower than the 1964 event. After 1983, only one flood is discussed: According to the 2019 HMP, the August 23, 2005 flood reached 2 to 3 ft above high tide and damaged property and infrastructure. A seawall was built in 1985 that may be reducing flooding from wave setup and runup.

In 1997, USACE used several HWMs to estimate the 1964 flood reached 18.8 ft MLLW. This is used for the FIRM 100-year base flood elevation (BFE). The elevation datum is questionable because Togiak does not have a MLLW datum connected to a benchmark. The 2010 FIRM report explains that the MLLW datum is 10.4 ft below NAVD 88, but the geoid is unspecified. USACE placed a HWM sign in Togiak but there is no published survey with a geodetic datum. A 2012 USACE survey with a stadia rod measured the sign to be 2.4 ft (0.73 m) above ground level. The 2003 DTM (generated from contours and converted to NAVD 88 [geoid12b]) shows the sign ground elevation is 4.18 m NAVD 88 (geoid12b), making the sign 4.91 NAVD 88 (geoid12b). The DTM vertical RMSE is 0.42 m (n = 7). When the FIRM was created in 2010, two common geoids in use were geoid96 and geoid99. Assuming the FIRM used either model, the BFE is 5.10 or 4.99 m NAVD 88 (geoid12b), respectively. These agree with the HWM sign estimate. Using the mean value, the BFE is approximately 5.0 m NAVD 88 (geoid12b). Uncertainty is the RSS of half the range of BFE estimates (0.1 m) and DTM error, resulting in 0.4 m. Togiak recently received a tidal datum that connects only NAVD 88, MHW, and MHHW (2.640 m above NAVD 88). The October 1964 flood is estimated to have reached 7.7 ± 1.3 ft MHHW (5.0 ± 0.4 m NAVD 88). A flood of this elevation would inundate most of the townsite with 1 to 3 ft of water, a major flood event matching the descriptions.

Toksook Bay

USACE flood reports and the Toksook Bay 2014 HMP indicate only minor flooding occurs. The record flood occurred October 28, 1995. The October 1995 Storm Data report states water reached 6.5 ± 1.0 ft “above normal.” The tidal range is 9.2 ft, so this is likely referring to a high water line like MHW or MHHW (0.4 ft difference). The October 28, 1995 flood is estimated to have reached 6.1 ± 1.1 ft MHHW (4.18 ± 0.34 m NAVD 88). Uncertainty is calculated as the RSS of the water elevation observation uncertainty and the difference of MHW and MHHW. This is also anticipated to be the record for Umkumiut.

Tuntutuliak

The storm surge flood of record in Tuntutuliak reached 2.0 ± 1.0 ft MHHW (3.4 ± 0.3 m NAVD 88) on August 17, 1990. This record was identified by USACE in 1996 through interviews with community members and a non-GPS survey. For the Community Profile Map, DCRA surveyed the flood estimate to -0.17 m NAVD 88 (GEOID99). This is 3.38 m NAVD 88 (GEOID12B). USACE estimated the flood reached approximately the ground elevation of the FAA building at the old airport. We surveyed this to be 3.42 ± 0.05 m NAVD 88. This verifies the DCRA survey and datum conversion. Uncertainty is estimated to be 1 ft, given there are limited details about the flood elevation sources.

USACE and other reports don’t specifically describe the impacts of the 1990 flood in Tuntutuliak, but other floods have caused major impacts. The August 1990 Storm Data report describes damage to the region: “The worst damage was to private property such as subsistence food, boats, motors, fishing nets, fishing camps, and homes.” The November 1979 storm flooded 8 structures in Tuntutuliak and may have rivaled 1990. The 2015 HMP does not describe the community-specific impacts of the several storms occurring since 1990. Tuntutuliak is also subject to ice jam flooding, which has exceeded storm surge elevations in neighboring Napakiak.

Tununak

The highest known flood in Tununak reached 6.5 ± 1.3 ft MHHW (4.3 ± 0.4 m NAVD 88) on November 8-11, 2011. This estimate is based on elevations of boardwalks, the seawall, and imagery of blocks of ice left near town^[30]^. In a written survey collected by USACE in November 1968, “a village elder stated that he could never remember the village flooding; however, tides have come almost into the village.” This description also matches the November 2011 event, indicating it is effectively the flood of record. This caused minor flooding.

Umkumiut

Umkumiut is the traditional fish camp for Nightmute and also used by residents of Toksook Bay. For this reason, it is not the subject of USACE or other flood investigations, nor does it have a HMP. The record flood for Toksook Bay (3 miles northeast) is also used for Umkumiut: the October 28, 1995 flood is estimated to have reached 6.1 ± 2.0 ft MHHW (4.18 ± 0.61 m NAVD 88). Uncertainty is increased to 2.0 ft because no flood observations were found for Umkumiute. Observations of structure locations and the DSM suggest this level of flooding would surround some structures but only cause minor flood impacts.

Unalakleet

Unalakleet is built on a spit with a maximum ground elevation of about 16 ft MHHW (6.5 m NAVD 88) south of the runway. Most structures are built on ground above 11 ft MHHW (5.0 m NAVD 88). Due to waves, floods reach higher on the ocean side than the sheltered backside. Flood studies indicate the spit has never been completely flooded. Original descriptions collected by USACE state the highest known flood in Unalakleet reached 14.6 ft MHHW (6.06 m NAVD 88) on November 5, 1965. Recent sources increased the estimate to 16.6 ft MHHW (6.67 m NAVD 88), but this value would inundate the entire spit, an event inconsistent with local observations. Delayed data from the December 1965 Storm Data report states twelve homes were damaged by flooding (up to 20 inches depth) or undercutting in November. Flooding of 14.6 ft MHHW would inundate the southwest area up to the shore-parallel berm in the center of the spit. This area is densely populated today, but the 1980 Community Profile Map shows there were only 10 to 20 residences, consistent with damage observations of 12 homes. There is not enough evidence to narrow the flood estimate, so the original value is used with a greater uncertainty range: 14.6 ± 2.0 ft MHHW (6.06 ± 0.61 m NAVD 88). The November 9, 2011 storm reached 13.4 ft MHHW (5.70 m NAVD 88) with wave setup, and 8.2 ft MHHW (4.11 m NAVD 88) without setup^[26]^. Assuming wave setup also added 5.2 ft to the record event, the flood estimate for the sheltered side of the community is 9.4 ± 2.0 ft MHHW (4.47 ± 0.61 m NAVD 88).

Adjusted record elevation estimate: In 1971, USACE interviewed residents who indicated the 1965 flood reached 16 ft MSL (14.6 ft MHHW). However, subsequent reports claim the 1965 event reached 18 ft MSL. This second estimate originated from a letter to USACE from DOT about installing an Alaska National Airspace System Interfacility Communications Systems site for the FAA. This adjustment is based on one statement that the 1965 flood was approximately 3 ft greater in depth than the 1974 flood. DOT explains that the height of the 1974 flood was 4 ft above grade at the Native Store and 4.33 ft above grade at the fish cannery. Both structures were at approximately 11 ft MSL, bringing the 1974 flood elevation to 15 to 15.33 ft MSL. Both structures were located on the exposed side of the spit and subject to wave setup. If the 1965 flood were 3 ft higher (18 ft MSL), water would cross the entire spit and result in depths of about 1 to 6 ft under every residence in Unalakleet. No accounts describe flooding of this extent, and the November 1965 Storm Data report states only twelve homes were flooded. It is more likely that the 1965 flood was 16 ft MSL as originally reported and the November 1974 flood was approximately 15 ft MSL as observed. The claim of 1965 being 3 ft deeper may have been inaccurate, observing a localized rise in water level in a different area than where the 1974 observations were made, or observing a localized rise in water level at the site of the 1974 observations that does not reflect the flood level for the majority of the spit.

Utqiaġvik

The highest known flood in Utqiaġvik reached 11.6 ± 1.0 ft MHHW (4.57 ± 0.30 m NAVD 88) on October 3, 1963^[31,32]^. This caused major flood impacts. The Storm Data report (delayed data published December 1963) explains that there were unusually strong onshore winds with gusts up to 80 mph and a high tide that produced 15-foot waves. Thirty structures were destroyed, half of which were homes. Salt water contaminated the freshwater lake used by the community for drinking water. Fathauer^[33]^ explains that the storm originated from Siberia and crossed the Arctic Ocean over an essentially ice-free fetch of 800 to 1000 miles. Water reached about 400 ft inland and lifted several houses.

Hume and Schalk^[31]^ compared observations to conclude the water level was likely 12 ± 1 ft MSL (11.6 ft MHHW). Water was 2 ft above the beach that crested at 10 ft MSL. Waves were approximately 10 ft as well. Hume and Schalk^[31]^ suggest this was a 200-year event given that it was the highest flood known to residents. They note the 1954 storm was the previous record with a elevation of 9 to 10 ft MSL. Lynch et al.^[34]^ use wind hindcasts to model these and other events, finding that such storms are more frequent than the flood history would suggest, but sea ice often prevents the storms from generating this level of surge.

Wainwright

The flood of record in Wainwright occurred on October 3, 1963. The 2015 HMP claims there was a surge of 11 to 12 ft that damaged several homes and buildings, flooding approximately 50% of the community. However, the source material contradicts these claims. In 1971, USACE collected written surveys of flood impacts. Multiple surveys indicate no structures have ever flooded and water only rose 4 ft (surge) with waves reaching the bluff. We surveyed the bluff along 8 profiles and found the top is 7.66 m NAVD 88 (SD = 2.43 m) and the toe is 3.85 m NAVD 88 (SD = 0.50 m). Water exceeded the toe but likely did not exceed 5.23 m NAVD 88 (1 SD below the mean bluff top elevation), placing the estimate at 4.54 ± 0.69 m NAVD 88. This is 11.5 ± 2.3 ft MHHW. This was a minor flood.

Wales

According to USACE flood investigations, the flood of record in Wales occurred on November 10, 1974. USACE investigations and the November 1974 Storm Data report describe flooding of low-lying property, water reaching main street, minor damage to the runway, but no flooding of homes. This constitutes a moderate flood. Based on community observations and the 1980 Community Profile Map contours, USACE estimated the flood reached the 10 to 14 ft contours. The 14 ft limit includes waves. The 1980 map vertical datum is “assumed sea level.” Wales only has a tidal range of 1.0 ft and the contours appear to be equal to the DSM and tidal datum MSL. The values estimated by USACE match described flood impacts. The 1974 flood is estimated to have reached 9.5 ± 2.0 ft MHHW (4.00 ± 0.61 m NAVD 88) with wave runup reaching 13.5 ± 2.0 ft MHHW (5.22 ± 0.61 m NAVD 88). The uncertainty is 2 ft because the flood estimates were made on 2 ft contours.

### **S3 Inconclusive estimates of record floods**

The record flood elevation could not be determined for the following communities. Where possible, the highest known flood category is estimated.

Emmonak

Emmonak is 10 miles inland from the coast on the Yukon Delta. The city has an extensive history of major ice jam flood disasters. The highest known ice jam flood occurred on May 29, 1989, and reached 5.4 m NAVD 88 (9.4 ft MHHW; based on conversion from DCRA measurement of USACE estimate). The 2008 and 2014 HMPs indicate Emmonak is impacted by storm surge events^[20]^, including November 11-13, 1974, August 18, 1978, October 18, 1987, and September 22, 2005. No specific details were found to estimate these, but only minor to moderate impacts are anticipated.

Nelson Lagoon

USACE investigations and the Aleutians East Borough 2021 HMP indicate that Nelson Lagoon has never been flooded. Water has reached near structures, but there is no record event listed. The lowest ground with structures is about 4 ft MHHW (3.5 m NAVD 88) and the seawall is approximately 6 ft MHHW (4 m NAVD 88). Storm surge flooding has likely not exceeded these elevations on the lagoon side of the community, causing only minor flooding.

Kwigillingok

The 2015 HMP and USACE investigation do not specify the highest known flood. The HMP notes approximately half of the residents moved to Kongiganak after the February 1966 flood. No specific information on flood impacts is provided so an estimate cannot be made. A flood in October 2012 was approximately 2 ft above ground at the landfill. The landfill in use at this time was east of town across the river, set on the tundra with a fence and no berm. A landfill with a berm connected to town was built after this event. The ground in the old landfill is 2.3 ± 0.5 ft MHHW. The 2012 flood estimate is 4.3 ± 1.1 ft MHHW (4.20 ± 0.34 m NAVD 88). The uncertainty is the RSS of 1.0 ft for the water elevation estimate and 0.5 ft for the DTM and average landfill ground elevation. The record flood was February 1966 but there is no specific information other than approximately half of the community decided to relocate to Kongiganak afterward due to the flood hazard. The 1966 flood may have exceeded the 2012 event, but there is not enough information to be certain.

Eek

The 2014 HMP states flooding does not impact Eek because the community is built on a high bluff above the floodplain.

Point Lay

The 2015 HMP states that while large storms reach Point Lay, water has not flooded the community.

Nuiqsut

The 2015 HMP excludes Nuiqsut from the list of communities at risk of storm surge flooding.

Dillingham

The 2016 HMP states the highest known storm surge flood reached 10 ft MHHW in November 1929. Dillingham is built on high bluffs, so the worst impact listed was lost boats, indicating a minor flood.

Egegik

Egegik is built on high bluffs. The 2019 HMP states the community is not impacted by flooding.

Gambell

Gambell is built on a broad spit on the northwest tip of Saint Lawrence Island in the Bering Sea. No DSM or lidar DTM was available. The 2004 Community Profile Map shows the beach ridge on the west side of the spit rises from 26 to 32 ft NGVD 1929. The 2012 HMP explains that the combination of storm surge and waves can flood the runway and have reached near the school but not flooded structures. Storms typically come from the west^[28]^. The 1980 Community Profile Map shows an estimated area at risk of flooding in a 100-year event that reaches the school area but not the inland residences. It is not clear which storm was the record event. Although storms have not damaged structures, they inundate the runway, scour sediments, and deposit sediments on the runway and elsewhere. The 2012 HMP states the community evacuated in 1969 for a particularly strong storm. The specifics of the evacuation were not indicated, and there is no listing of damaged structures. For the period of 1985 to 2012, Erikson et al.^[28]^ model wave runup reaching 26.5 ft MHHW (9.0 m NAVD 88) in October 2000 and November 2011, and 27.1 ft MHHW (9.2 m NAVD 88) October 28, 1996. The record event likely exceeded these events but did not exceed the ground elevation near the school, approximately 30 ft MHHW (10 m NAVD 88). The community appears to be subject to major storms.

Ivanof Bay

USACE investigations indicate there is no history of storm surge flooding in Ivanof Bay. There is no HMP, and no Storm Data reports were found to describe flooding in Ivanof Bay.

Naknek

Naknek and South Naknek are built on high bluffs. The 2017 HMP describes minor impacts of flooding, including the loss of boats and destruction of low-lying property, but no inundation of structures. Sources do not identify a record flood.

Perryville

The 2019 HMP and USACE investigations agree that only minor flooding occurs at Perryville due to a combination of rainfall, high tides, and/or storm surge. Low-lying roads can become inundated and water has reached near the first floor of low-lying residences. The date of the record event is not listed.

Pilot Point

The 2019 HMP and USACE investigations agree that only minor flooding occurs at Pilot Point. Flooding can occur from storm surge or river ice jams. The barge landing and bulkhead can become flooded, as well as the roads to access them and surrounding subsistence fishing sites. Floods can deposit sea ice onto the roads, delaying access unless the debris is cleared. The highest known flood is not identified.

Port Heiden

Flooding in Port Heiden is overshadowed by the substantial erosion history. Currently, most of the community is situated far inland where coastal flooding does not reach. The road to the school is occasionally flooded by storm surge, preventing travel (personal communication, Jaclyn Christensen, May 2021). Storms cause minor flood impacts, which can include damage to boats and subsistence equipment if left in low-lying areas. The record event and elevation is not known.

Saint George

USACE investigations and the 2015 HMP indicate storm surge flooding does not impact Saint George.

Savoonga

The 2012 HMP and 2000 Economic Development Plan explain that Savoonga experiences erosion from storm surge, but the community is built on a bluff above the floodplain. Flood years of 2003, 2004, and 2005 are listed but no record event is discussed. Flooding is limited to minor impacts.

Twin Hills

The 2019 HMP explains that Twin Hills is situated on a bluff above the floodplain. Flooding reaches low-lying areas and can wash out Beach Road, reducing access to the landfill, cannery, and subsistence areas. Twin Hills experiences minor flooding, and no record event is listed.

### **S4 Infrastructure statistics**

Small structures like sheds were not delineated for most locations. Including them would introduce bias, where communities with small structures delineated would have many more structures than an equal community without small structures delineated. Small structures were filtered to reduce bias. To determine the area threshold to filter small structures, structure types are compared (Supplementary Figure 2). In communities where small structures were delineated, unspecified structures have a median area of 13 m^2^ and 97.5% are less than 56 m^2^. Residences have a median area of 106 m^2^ and 75% of identified residences are greater than 73 m^2^. Although the dataset shows 25% of residences have relatively low area, a closer examination found that many of the smaller structures labeled “residence” (approximately less than 40 m^2^) are actually sheds that were mislabeled. Several commercial structures are below the 56 m^2^ threshold. These are typically fuel tanks and should not be equated to the target structures. Most structures greater than 56 m^2^ are residences, and 29% are not identified but likely mostly residences (Table 3).

Table 3. Number and percentage of structures greater than 56 m^2^ in the study.

| **Structure Type** | **Count** | **Percent of Total** |
| --- | --- | --- |
| Not Identified | 2678 | 29% |
| Residential | 5277 | 57% |
| Public | 912 | 10% |
| Commercial | 406 | 4% |
| **Total** | **9273** | **100%** |

The elevation of a structure’s first floor is a commonly used metric for flood risk management. Few communities have first floor elevation datasets, so the only available information is ground elevation. Structures are elevated on pilings in many of the study sites, so the first floor is higher than ground level. The height above ground level (AGL) varies for each structure. To determine the reliability of using ground elevation as a proxy for structure elevation, we examine the first-floor elevations of 4 communities where these data exist: Alakanuk, Deering, Kipnuk, and Kotlik (Table 3; first floor elevation data provided by DGGS Coastal Hazards Program). Lidar DTMs are available for these locations. The mean first-floor elevation for all structures is 1.58 m AGL (SD = 0.58 m; Table 4). Structures at lower elevation would need to be built higher above ground to evade flooding. This potential bias is evaluated by comparing ground elevation to first-floor elevation (Supplementary Figure 3). There is a mild trend of structures being built higher when at lower elevation, but the trend varies across communities. Ultimately, inferences can be made about the likely range of first floor elevations, but the variability is large, meaning this generalization cannot be applied to specific communities.


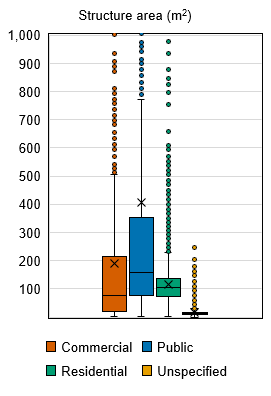


Supplementary Figure 2. Box plot of the area (m^2^) of different structure types.

The centerline of the quartile boxes is the median area. The X is the mean. Circles are outliers, lying greater than 1.5 times the interquartile range. Most identified structures have areas of at least 70 m^2^. Unspecified structures have a median area of 13 m^2^ and 97.5 percent are below 56 m^2^. Commercial structures include numerous fuel tanks with relatively small area. Data for commercial, public, and residential structures come from 41 communities. Unspecified structure data comes from 10 communities. The Y-axis is limited to 1000 m^2^ to highlight the comparisons being made, but outliers extend up to 15,000 m^2^.

Table 4. Mean first floor elevations above ground level (AGL) in four communities

individually and combined. All are found to be normally distributed using the Kolmogorov-Smirnov test.

| **Community** | **n** | **Mean Height AGL (m)** | **SD (m)** |
| --- | --- | --- | --- |
| Alakanuk | 200 | 1.72 | 0.55 |
| Deering | 68 | 1.40 | 0.62 |
| Kipnuk | 213 | 1.67 | 0.59 |
| Kotlik | 170 | 1.40 | 0.52 |
| Combined | 651 | 1.58 | 0.58 |

Supplementary Figure 3. Comparison of structure elevation AGL vs. ground elevation.

In general, structures on higher ground are not built as high above the ground. This trend is strongest in Alakanuk (teal). The low R^2^ values indicate a linear trend does not describe this relationship well.

The communities in the study area place considerable reliance on subsistence-based activities for food collection and storage for which no datasets exist to quantify. Subsistence activities require vehicles such as boats, light utility vehicles (colloquially called four-wheelers), light trucks, and snowmobiles (colloquially called snowmachines). There are also shed-style structures for treating food and storage often handmade of basic construction material and/or driftwood. In addition, many communities have boardwalk systems and utility corridors (metal housing surrounding utility pipelines placed on or raised slightly above the ground). While datasets were not available to specifically quantify subsistence assets in every community, these assets (except boats) are most often located adjacent to structures and are intrinsically represented by structure location. By reviewing guidelines from the U.S. Federal Emergency Management Agency (FEMA)^[35]^ and vehicle schematics, we estimated that flooding of 0.6 m totals snowmobiles (i.e., the damage is so severe that cost of repairs is more than the vehicle is worth) ; 0.9 m totals light utility vehicles and cars; and 1.2 m totals light trucks.

### **S5 Relative sea level rise projections**

RSLR projections from Sweet et al.^[36]^ are averaged for all nodes near each jurisdiction (Supplementary Figure 4). In general, RSL is projected to rise faster in the northern part of the study. Sweet et al.^[36]^ estimate the likelihood of the 0.5 m GMSL by 2100 scenario is 50 to 99% depending on global average temperature. The estimated likelihood of the 1.0 m scenario is 5 to 23%. The difference between the scenarios increases over time due to the increased dependence on diverging possible futures of greenhouse gas emissions and associated global warming. Scenario 0.5 m projects relatively consistent SLR rates, whereas scenario 1.0 m projects faster rates over time. A user can compare the exposure results to RSLR projections to gauge potential future exposure under the two presented emission scenarios. To apply RSLR to the previously listed flood results, the user must subtract the RSLR value from the elevation.

Supplementary Figure 4. Average RSLR from 1992 (the current tidal datum) to 2100 for each jurisdiction

under GMSL scenario 0.5 m by 2100 (top) and 1.0 m by 2100 (bottom). Data from Sweet et al.^[36]^.

## **References**

1. OCM Partners. 2019 USACE NCMP topobathy lidar DEM: Alaska. (2023).

2. Quantum Spatial. *North Slope Borough communities, Alaska 3DEP LiDAR technical data report*. 39 (2019).

3. Overbeck, J. R., Hendricks, M. D. & Kinsman, N. E. M. *Photogrammetric digital surface models and orthoimagery for 26 coastal communities of western Alaska*. 3 https://doi.org/10.14509/29548 (2016).

4. Overbeck, J. R., Hendricks, M. D. & Kinsman, N. E. M. *Photogrammetric digital surface models and orthoimagery for the continuous coastline, Wales to Platinum, Alaska*. 21 https://doi.org/10.14509/29744 (2017).

5. Glenn, R. J., Overbeck, J. R. & Heim, R. *Color indexed elevation maps for flood-vulnerable coastal communities in western Alaska*. 4 https://doi.org/10.14509/30160 (2019).

6. U.S. Geological Survey. *AK Yukon Kuskokwim QL2 lidar: Airborne LiDAR report*. 22 (2017).

7. Southerland, L. E. & Kinsman, N. E. M. *Lidar data for the community of Golovin, Alaska*. 23 http://dx.doi.org/10.14509/29127 (2014).

8. Herbst, A. M. & Daanen, R. P. *High-resolution lidar data for Kotlik, Western Alaska*. 7 https://doi.org/10.14509/30561 (2020).

9. AeroMap U.S. *Lidar final report AK0301 - Shishmaref*. 176 (2006).

10. Bogardus, R. C. Identifying spatial patterns of storm driven flooding and erosion at Nelson Lagoon, Alaska. (University of Alaska Fairbanks, 2021).

11. Buzard, R. M., Christian, J. E. & Overbeck, J. R. *Photogrammetry-derived orthoimagery and elevation for Saint Paul, Alaska, collected July 22-25, 2021*. 7 http://doi.org/10.14509/30836 (2022).

12. Buzard, R. M., Overbeck, J. R., Chriest, J., Endres, K. L. & Plumb, E. W. *Coastal flood impact assessments for Alaska communities*. 16 https://doi.org/10.14509/30573 (2021).

13. Buzard, R. M., Overbeck, J. R., Miller, K. Y. & Christian, J. E. *Coastal flood impact assessments for Alaska communities: Alakanuk*. 41 https://doi.org/10.14509/30825 (2022).

14. Trudeau, C. Chefornak bands together to combat climate change. *KYUK* https://alaskapublic.org/2018/01/25/chefornak-bands-together-to-combat-climate-change/ (2018).

15. Buzard, R. M. & Overbeck, J. R. *Coastal flood impact assessments for Alaska communities: Hooper Bay*. 23 https://doi.org/10.14509/30856 (2021).

16. Buzard, R. M. & Overbeck, J. R. *Coastal flood impact assessments for Alaska communities: Golovin*. 32 https://doi.org/10.14509/30855 (2021).

17. Buzard, R. M., Maio, C. V., Verbyla, D., Kinsman, N. E. M. & Overbeck, J. R. Measuring historical flooding and erosion in Goodnews Bay using datasets commonly available to Alaska communities. *Shore & Beach* **88**, 11 (2020).

18. Chapman, R. S., Kim, S.-C. & Mark, D. J. *Storm-induced water level prediction study for the western coast of Alaska*. 92 (2009).

19. Wise, J. L., Comiskey, A. L. & Becker Jr., D. *Storm surge climatology and forecasting*. 32 (1981).

20. Buzard, R. M., Overbeck, J. R., Turner, M. M. & Christian, J. E. *Coastal flood impact assessments for Alaska communities: Kotlik*. 57 https://doi.org/10.14509/30783 (2022).

21. Kotzebue cleans up damage from flood. *Anchorage Daily News* 1 (1990).

22. Buzard, R. M., Overbeck, J. R., Miller, K. Y. & Christian, J. E. *Coastal flood impact assessments for Alaska communities: Napakiak*. 45 https://doi.org/10.14509/30782 (2021).

23. Buzard, R. M., Overbeck, J. R. & Miller, K. Y. *Coastal flood impact assessments for Alaska communities: Nunam Iqua*. 31 https://doi.org/10.14509/30675 (2021).

24. Kindle, E. M. Notes on the Point Hope Spit, Alaska. *The Journal of Geology* **17**, 178–189 (1909).

25. Buzard, R. M., Turner, M. M., Miller, K. Y., Antrobus, D. A. & Overbeck, J. R. *Erosion exposure assessment of infrastructure in Alaska coastal communities*. RI 2021-3 http://www.dggs.alaska.gov/pubs/id/30672 (2021) doi:10.14509/30672.

26. Kinsman, N. E. M. & DeRaps, M. R. *Coastal hazard field investigations in response to the November 2011 Bering Sea storm, Norton Sound, Alaska*. 51 https://doi.org/10.14509/24484 (2012).

27. U.S. Army Corps of Engineers. *Shaktoolik coastal flooding analysis*. 73 (2011).

28. Erikson, L. H., McCall, R. T., van Rooijen, A. & Norris, B. *Hindcast storm events in the Bering Sea for the St. Lawrence Island and Unalakleet regions, Alaska*. 47 http://dx.doi.org/10.3133/ofr20151193 (2015).

29. USACE. *Flood damage reduction: Stebbins, Alaska*. 27 (1988).

30. Coastal Villages Region Fund. Fall 2011. *Neqsurtet Nepiit: The sound of the fishermen* vol. 13 27 (2011).

31. Hume, J. D. & Schalk, M. Shoreline processes near Barrow, Alaska: A comparison of the normal and the catastrophic. *Arctic* **20**, 86–103 (1967).

32. Lantz, T. C., Moffat, N. D., Jones, B. M., Chen, Q. & Tweedie, C. E. Mapping exposure to flooding in three coastal communities on the North Slope of Alaska using airborne LiDAR. *Coastal Management* **48**, 96–117 (2020).

33. Fathauer, T. F. *A forecast procedure for coastal floods in Alaska*. 27 (1978).

34. Lynch, A. H., Lestak, L. R., Uotila, P., Cassano, E. N. & Xie, L. A factorial analysis of storm surge flooding in Barrow, Alaska. *Monthly Weather Review* **136**, 898–912 (2008).

35. FEMA. *Hazus 5.1 flood model technical manual*. 110 (2022).

36. Sweet, W. V. *et al.* *Global and regional sea level rise scenarios for the United States: Updated mean projections and extreme water level probabilities along U.S. coastlines*. 111 https://oceanservice.noaa.gov/hazards/sealevelrise/noaa-nostechrpt01-global-regional-SLR-scenarios-US.pdf (2022).
